# Genotype-Based Housing as a Potential Confounder in Studies Using Transgenic Mouse Models—Insight from the A53T Mouse Model of Parkinson’s Disease

**DOI:** 10.3390/biomedicines13061506

**Published:** 2025-06-19

**Authors:** Olga Dubljević, Miodrag Dragoj, Milica Potrebić Stefanović, Maja Srbovan, Miloš Stanojlović, Željko Pavković

**Affiliations:** 1 Department of Neurobiology, Institute for Biological Research “Siniša Stanković”—National Institute of Republic of Serbia, University of Belgrade, 11060 Belgrade, Serbia; miodrag.dragoj@ibiss.bg.ac.rs (M.D.); milica.potrebic@ibiss.bg.ac.rs (M.P.S.); maja.srbovan@ibiss.bg.ac.rs (M.S.); milos.stanojlovic@ibiss.bg.ac.rs (M.S.); zeljko.pavkovic@ibiss.bg.ac.rs (Ž.P.); 2 Department of Pharmacology, Toxicology and Pharmacy, University of Veterinary Medicine Hannover, Foundation, 30559 Hannover, Germany

**Keywords:** transgenic animal models, A53T, genotype-based housing, memory impairment, Parkinson’s disease, translation, reproducibility

## Abstract

**Background/Objectives**: Environmental factors, including the differences in genotype-based housing (GbH), can act as confounding variables in studies using transgenic mouse models, potentially influencing experimental outcomes and limiting their reproducibility and translational value. Despite the widespread use of transgenic models in preclinical studies, the extent to which housing conditions can affect the behavioral and molecular parameters of interest remains poorly understood. This study aims to investigate how different GbH conditions influence visuo-spatial memory and gene expression in the A53T mouse model (JAX006823) of Parkinson’s disease (PD) during the pre-motor phase. **Methods**: A53T+ transgenic male mice and their non-transgenic littermates (A53T−) were housed in either mixed-genotype (MGH) or single-genotype (SGH) environments from postnatal day (PND) 30, with C57BL/6J mice serving as the controls. A behavioral assessment using the Novel Object Recognition and Object Location Tests was conducted at PND 180, followed by a qPCR analysis of *Iba1*, *Gfapα*, *Bdnf*, *Tnfα*, *Il-1β*, and *Il-6* expression in the medial prefrontal cortex and the hippocampus. **Results**: The variations in GbH influenced behavior and mRNA expression differently in the A53T+ and A53T− animals. Specifically, the A53T− mice in SGH environments displayed behavioral and molecular profiles similar to the C57BL/6J controls, while the same was not evident in the MGH environments. In the A53T+ mice, the mRNA expression of *Iba1, Gfapα, Bdnf,* and *Tnfα* was sensitive to variations in GbH, while memory impairment was not. **Conclusions**: This study highlights the importance of considering environmental factors in studies using transgenic animal models. The obtained data suggests that GbH can influence the parameters of interest in preclinical research, implicating the need for the optimization of future study designs.

## 1. Introduction

Transgenic mouse models remain essential tools in biomedical research, particularly for studying the pathophysiology of complex human diseases such as neurodegenerative disorders [1,2]. However, growing evidence suggests that the experimental outcomes in studies using these models can be potentially influenced by the animals’ microenvironment, including social housing conditions, cage composition, handling, and broader environmental variables [3]. These factors, often overlooked when designing studies, can potentially interact with genetic background to impact the behavioral, physiological, and molecular outcomes in unpredictable ways. Despite the efforts to standardize protocols (e.g., in [4]), small differences in housing conditions across studies may contribute to inconsistencies in findings, impacting both the reproducibility of pre-clinical results and their translatability to clinical contexts [3].

There are currently no official guidelines on whether transgenic carriers and non-transgenic littermates, which are often used as controls, should be housed in mixed-genotype environments (MGH) or single-genotype environments (SGH). Certain authors recommend housing transgenic carriers with controls to reduce the confounding variability [5]; however, the effect of a shared microenvironment on these animals remains unclear. The differences in their inherent behavioral phenotype, e.g., one group being more socially dominant or aggressive than the other [4], could potentially influence the behavior of both groups, as well as the parameters related to stress [4,6,7,8] and/or even inflammation [8]. Additionally, considering that neurobiological processes can be altered through the brain–gut axis [9,10], co-housing healthy animals along with those genetically altered to model neurodegenerative diseases (with potential dysbiosis) may affect both phenotypes, potentially compromising the validity of preclinical research. If phenotype expression can be sensitive to environmental factors, overlooking these variables could lead to certain parameters of disease being masked or exaggerated. Addressing genotype-based housing (GbH) as a potential confounding factor is, therefore, critical for improving the standardization of study designs and ensuring that animal models remain reliable tools for translational research. To address the potential effect of co-housing transgenic animals with controls, we used the Hualpha-Syn (A53T) transgenic mouse line G2-3 (JAX006823 strain) [11], a promising model to be used in preclinical studies of pre-motor symptoms of Parkinson’s disease (PD). PD is a complex neurodegenerative condition marked by motor symptoms as well as non-motor manifestations which can precede the onset of motor deficits by years [12,13,14]. These pre-motor symptoms, including cognitive impairments, mood disturbances, and autonomic dysfunctions [15,16], are critical for early diagnosis [17], patient quality of life, and the development of valid animal models for studying PD [18]. Cognitive impairment, particularly in the domains of visuo-spatial memory and executive functioning, is a prominent feature of pre-motor PD pathology with 20–30% of patients presenting mild cognitive impairment (MCI) at the time of PD diagnosis, and many patients who do not meet the official criteria for MCI report subjective cognitive complaints [19,20]. Unfortunately, the lack of animal models that exhibit clinically relevant pre-motor symptoms of PD, such as spontaneous short-term visuo-spatial memory impairment, dysbiosis, and constipation, remains an ongoing issue for the field. The hemizygous A53T mice (A53T+) spontaneously develop constipation [21] and olfactory dysfunction [22] in the pre-motor phase of the disease which is a rare feature for a mouse line that is designed primarily as a model for synucleinopathies. The mice overexpress human α-Synuclein under the control of the murine prion promotor and develop motor impairments after 12 months of age [23], making them particularly suited for investigating the early pre-motor symptoms of PD. Long-term memory dysfunction was also reported in the pre-motor phase [23], although short/working visuo-spatial memory was never assessed.

The molecular mechanisms underlying the non-motor symptoms of PD remain unclear with neuroinflammation being linked to symptoms such as cognitive decline in PD [24], and elevated serum levels of IL-6 and *TNFα*/TNFα correlating with non-motor symptom severity [25,26,27]. The hippocampus (HPP), relevant to cognition, is a possible site of PD-related cognitive decline, potentially mediated by IL-1β [28]. While IL-1β supports learning under normal conditions, its elevated levels impair cognition [29,30], and blocking it is shown to improve cognitive function in certain cases [31]. Increased *IL-1β* expression is also observed in the frontal cortex of post-mortem PD patients [32], a region involved in cognition (especially executive functions) and affected in MCI [33]. Neuroinflammation also influences neurotrophin production, particularly BDNF [34], which is implicated in the non-motor symptoms in PD [35,36]. PD patients show reduced BDNF levels in serum and cerebrospinal fluid (CSF), correlating with poorer cognitive performance [37,38]. However, increased CSF BDNF levels in different stages of PD may reflect glial compensatory responses [35,39].

Understanding the limitations and translatability of in vivo models is crucial for the broader aim of translational research, i.e., bridging the gap between preclinical/basic research and clinical implementation [40,41]. Considering the fact that short-term memory deficits seem to be a potentially significant subject of research in this PD model, this study aims to investigate how the GbH influences this feature, as well as the gene expression of parameters relevant to PD, in order to refine it, as well as other preclinical models like it, with the broader goal of enhancing their translatability and improving the designs of future preclinical studies. In view of this broader aim, the specific objectives of this study were as follows: (1) to evaluate the potential of the Hualpha-Syn(A53T) transgenic mouse line G2-3 (JAX006823 strain) to model pre-motor short-term visuo-spatial memory deficits related to PD; (2) to assess the mRNA expression of microgliosis (*Iba1*) and astrogliosis (*Gfapα*) as well as of proinflammatory cytokines associated with the non-motor symptoms of PD (i.e., *Tnfα*, *Il-1β,* and *Il-6*) in the medial prefrontal cortex (mPFC) and HPP of the mice, as well as the mRNA expression of *Bdnf* in the same structures; (3) to evaluate the critical impact of GbH on all aforementioned pre-motor parameters associated with PD in A53T+ and A53T− animals.

In addition to the main experimental comparisons, a cohort of wild-type C57BL/6J mice (used for backcrossing and maintaining the colony) was included in the assessment in order to address another methodological consideration associated with this model. Specifically, the Jackson Laboratory (i.e., the producer) states that both non-transgenic littermates and C57BL/6J mice may be used as controls equally in this model (https://www.jax.org/strain/006823 last accessed on 14 June 2025). However, we wanted to treat this assumption as a variable of interest, as a lack of standardization in control selection may impact how the results from different studies using this model are interpreted.

## 2. Materials and Methods

### 2.1. Ethical Statement

All interventions and procedures were performed in compliance with the Serbian and European Animal Welfare Act. The overall study design was approved by the corresponding authority in Belgrade, Serbia (the Ethical Committee of the Institute and by the National Ethics Research Committee; Ethical decision number: 323-07-02149/2023-05; decision valid from 3 October 2023; document available in the Supplementary Section). The mice were handled in accordance with the European Directive 2010/63/EU on the protection of animals used for scientific purposes [42].

### 2.2. Animals and Housing Conditions

A total of 40 male mice were used for the purpose of this study: A53T transgenic mice (*n* = 16; second and third generation of in-house breeding), their non-transgenic littermates (*n* = 16; second and third generation of in-house breeding), as well as C57BL/6J (obtained from The Jackson Laboratory) which served as additional controls (*n* = 8). By including an additional control group, we aimed to clarify whether C57BL/6J and non-transgenic littermates are truly interchangeable in behavioral and molecular outcomes.

The colony of A53T mice was maintained by backcrossing transgenic males to C57BL/6J females, as per the recommendations provided by the producer, using “trio breeding groups”, consisting of one male and two females who are reproductively viable, as per the NIH and Jackson Laboratory guidelines (https://www.jax.org/jax-mice-and-services/customer-support/technical-support/breeding-and-husbandry-support/colony-planning last accessed on 14 June 2025). Once visually pregnant, females were placed in a separate cage with nesting material and housed separately until the litter was weaned (postnatal day, PND 21–24). The genotype of the offspring (carriers, A53T+; non-carriers, A53T−) was revealed by PCR analysis of the DNA (obtained from the tip of tail) at the time of weaning. The final placement in designated groups/cages was performed following genotype analysis (PND 29 ± 2; see the Procedure Section of the manuscript for further details). The experimental groups were established in this particular timeframe, based on evidence that, beyond early postnatal life, the period between weaning and young adulthood represents another critical window for modulating microbiome composition [43]. The mice were housed in stable social groups of four per cage (Type 1L cages, 405 cm^2^, ZOONLAB, Castrop-Rauxel, Germany), and cage composition remained unchanged throughout the experiment. Group housing was implemented prior to sexual maturation to minimize male aggression [44]. The C57BL/6J mice were maintained under identical housing conditions but were not co-housed with either A53T+ or A53T− mice. All animals were housed in the following conditions: a room temperature maintained at 22 ± 1 °C, relative humidity at 50 ± 5%, and a 12: 12 h light/dark cycle. The light, provided in a diffuse manner at a level of 20–50 lux, was turned on at 7 a.m. and off at 7 p.m. The standard sized cages were made from transparent plastic. Autoclaved wood shavings (PREMIUMSPAN^®^, HVT Hobelspanverarbeitung GmbH, Dittersdorf, Germany) were provided in sufficient quantity to cover the floor to a depth of 2 cm as bedding material. To minimize stress and aggression, a portion of used bedding was intentionally left in the cage during each cage change to preserve familiar olfactory cues and animals were regularly monitored by staff using a questioner for monitoring stress and aggression (available in Supplementary Section). The diet consisted of commercial pellets (produced on demand by Gebi Doo, Cantavir, Serbia) with 20% protein, 5% fat, and 5.5% fiber, and tap water was available *ad libitum*. Environmental enrichment measures were also implemented in the form of plastic and cardboard tubes as well as nesting material, to minimize stress and aggression [45]. The nesting material used was provided by our facility (i.e., produced in vivarium at Institute for Biological Research “Siniša Stanković”) and consisted of sterilized (120 °C) shredded paper strips, in accordance with [46]. The mice were 6 months old at the beginning of the behavioral assessment, after which they were ethically euthanized (via cervical dislocation). Seeing as the goal was to evaluate the potential of this model to replicate the pre-motor features of PD, and that previous studies have shown that certain non-motor symptoms relevant to PD, such as cognitive impairments and certain molecular changes, can begin to emerge in A53T mice from as early as 5 months of age [23], 6 months was selected as a suitable time point, as it reflects a stage when the animals are fully adult but still well before the onset of motor dysfunction. This allowed us to isolate and examine the pre-motor behavioral and molecular phenotypes without the confounding influence of neurodegeneration or motor decline, thus aligning with the scope and aims of our study.

### 2.3. Procedure

**Figure 1:**
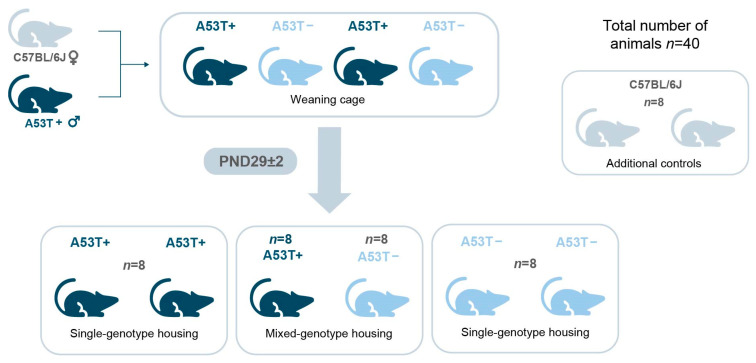
Animal weaning and segregation into SGH and MGH. The colony of A53T mice was maintained by backcrossing transgenic males to C57BL/6J females. A53T+ and A53T− animals were weaned in the same cage, until PND 29 ± 2 when they were segregated into SGH or MGH. C57BL/6J animals were kept in SGH as additional controls. Mice were housed in stable social groups of four per cage.

**Figure 2:**
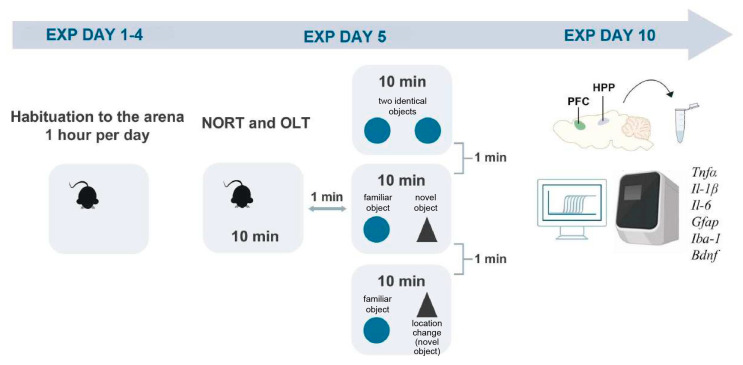
Graphical representation of the procedure. All animals were habituated to the testing arena from experimental day 1−4; on experimental day 5 after a 10 min habituation period to the testing arena, NORT and OLT were performed to assess short-term memory. On experimental day 10, the mPFC and HPP of animals were extracted and stored at −80 °C for subsequent qRT-PCR analysis of the expression of relevant inflammatory and neurotrophic markers (i.e., *Iba1*, *Gfapα*, *Il-6*, *Il-1β*, *Tnfα,* and *Bdnf*).

At PND 29 ± 2, the A53T transgenic male mice (A53T+) and their non-transgenic littermates (A53T−) were divided into two different groups: those kept in mixed-genotype housing (MGH, i.e., A53T+ and A53T− animals sharing a home cage) and those kept in single-genotype housing (SGH; A53T+, and A53T− animals in cages segregated by genotype; see Figure 1). Behavioral testing was performed on 6-month-old male mice. Short-term memory assessment was conducted via the Novel Object Recognition test (NORT) along with the Object Location Test (OLT) [47]. Animals were sacrificed via cervical dislocation 5 days after the testing, between 11 AM and 1 PM, to reduce the effect of behavioral assessment on the parameters of interest, and the mPFC and HPP were isolated and stored at −80 °C for subsequent RT-PCR analysis. These regions were selected because they are consistently implicated in the pathophysiology of PD, particularly in relation to cognitive deficits associated with the disease [28,32,33]. The mRNA expression of relevant proinflammatory cytokines (*Il-1β*, *Il-6,* and *Tnfα*), well-established markers of microglia/macrophage (*Iba1*) and astrocyte (*Gfapα*) activation, as well as of the brain-derived neurotrophic factor (*Bdnf*) were evaluated. The sequence of events described above is visible in Figure 2.

### 2.4. Behavioral Assessment of Short-Term Visuo-Spatial Memory

A detailed description of the Novel Object Recognition Test has been provided in our earlier work [48], with specific modifications made to better suit the requirements of this study and the mice used. Behavioral assessments were conducted daily between 10 AM and 4 PM by a female experimenter. One person preformed the testing to ensure consistency. Three animals were tested simultaneously using three independent monitoring systems. To minimize variability, a maximum of three sets (of three animals) were tested per day, with balanced placement across the time of day and monitoring system used. As the animals reached 6 months of age at different times, the testing schedule was spread over 3 weeks. The specific testing day for each animal was based on its age, and the order of testing within and across groups was randomized using a predefined schedule created by an independent experimenter. Animals were habituated to the testing chamber 30 min before being exposed to the test. All animals were tested within the same chamber and only one researcher was responsible for handling the animals and conducting the assessment. To evaluate short-term visuo-spatial memory, the NORT with an additional phase requiring spatial memory (OLT) was performed [47,49]. The animals were habituated to the arena for 4 days (1 h per day). On the fifth day, after a 10 min habituation period, two identical objects were introduced to the arena in Phase I of the NORT; in Phase II, one of the objects was replaced with an object with different visual properties, i.e., the novel object. The order of object replacement, i.e., whether the right or left object was replaced, was counterbalanced between mice. In Phase III, i.e., OLT, the now familiar object was placed in a different location. Each phase lasted for 10 min to ensure enough time for learning while the pauses between phases lasted 1 min to ensure that short-term/working memory was assessed [50,51]. Time was considered exploratory only when the mice touched the object with their noses or oriented their noses toward an object within 2 cm. Turning away from the object was not count as exploratory behavior. Mice were prevented from sitting on the object due to its design (familiar objects: two identical egg-shaped objects 8 cm high and 5 cm wide; the novel object, a cone-shaped object that had a similar size). All behavior was recorded and subsequently analyzed by two independent researchers. To minimize observer bias, individuals conducting quantification for the NORT and OLT were blinded to genotype, and mice were identified solely by ear markings throughout the experiment. Behavioral testing followed a predetermined schedule that balanced the side on which the novel object appeared. Genotypes were revealed only after all analyses were completed. A codebook of ear identification markers is provided in the Supplementary Material. The preference index was calculated using the following formula:

(1) $$\mathrm{Discrimination}\;\mathrm{Index}\;\mathrm{based}\;\mathrm{on}\;\mathrm{Number}\;\mathrm{of}\;\mathrm{Approaches}=\frac{Number\;of\;times\;the\;novel\;object\;was\;approached}{Total\;number\;of\;approaches\;(both\;objects)}$$

(2) $$\mathrm{Discrimination}\;\mathrm{Index}\;\mathrm{based}\;\mathrm{on}\;\mathrm{Exploration}\;\mathrm{Time}=\frac{Time\;spent\;exploring\;the\;novel\;object\;(seconds)}{Sum\;of\;time\;spent\;exploring\;both\;objects\;(seconds)}$$

The obtained Discrimination Index (DI) values are compared with the level of random exploration (for which the value index of 0.5 is used) using a one-sample *t*-test, where the absence of a significant difference indicates a lack of preference for the novel object, while a significant difference indicates the presence of a preference for the novel object. In the NORT, a significant discrimination index shows a preference for the novel object, suggesting good memory and cognitive function of the experimental subjects, while in the OLT, a significant discrimination index reflects the animal’s ability to remember spatial relationships by favoring exploration of the relocated object, demonstrating an effective spatial memory [49].

### 2.5. Quantitative Real-Time Reverse-Transcription PCR Analysis

Total RNA was isolated from mPFC and HPP tissue. The isolation was carried out using the Trizol^®^ reagent (Invitrogen Life Technologies by Thermo Fisher Scientific, Waltham, MA, USA, 15596026) with the addition of chloroform (100 µL per 500 µL of Trizol) according to the manufacturer’s instructions. After shaking and centrifugation (12,000 *g*/15 min/4 °C), the supernatant was collected, and RNA was precipitated by isopropyl alcohol and centrifugation (12,000 *g*/10 min/4 °C). Harvested RNA was washed twice with 75% ethanol and resuspended in RNAse-free water. Genomic DNA residue was eliminated from the samples using a DNase I, RNase-free kit (Thermo Scientific, EN0521), after which cDNA was synthesized using a High-Capacity cDNA Archive Kit (Applied Biosystems, Waltham, MA, USA, 4374966), according to the manufacturer’s instructions. qRT-PCR was performed using a Maxima SYBR Green/ROX qPCR Master Mix (Applied Biosystems, K0222) and QuantStudio 3 RT-PCR system (Applied Biosystems). The primer sequences are presented in Table 1. Target gene expression levels were determined by the comparative 2^-(delta C_t_) quantification method using *Gapdh* as the reference gene.

### 2.6. RNA Sequencing and Related Data Analysis

To demonstrate A53T+ animals as a suitable model for PD, the total RNA of 5 representative animals from all groups was extracted from hippocampal tissue and sent to Novogene (GRAD) for library preparation and sequencing. Libraries were prepared using the Eukaryotic mRNA-seq Directional protocol and sequenced on the NovaSeq X Plus platform, generating 150 bp paired-end reads with approximately 6 Gb output per sample. The resulting raw data (150 bp paired-end reads) were provided in FASTQ format. Quality control of raw reads was performed using FastQC v0.12.1 to assess base quality scores, duplication levels, adapter content, and other quality metrics [52]. A STAR-featureCounts-DESeq2 workflow was utilized for the alignment of the sequence reads (FASTQ format) to the reference genome assembly (January 2020 GRCm39/mm39), quantitation, and differential expression analysis [53,54,55,56]. To enable the detection of the human transgene, the human mutated alpha-synucleine (SNCA) canonical transcript (NM_000345.4) was added to the GRCm39 (mm39) mouse reference genome. A corresponding GTF annotation entry was created. A STAR genome index was generated using STAR v2.7.11b with the custom reference FASTA and modified GTF. Reads were aligned to the custom mouse + SNCA reference genome using STAR v2.7.11b. Paired-end reads were mapped with default settings optimized for directional RNA-seq data. Aligned reads were quantified using featureCounts v2.1.1 from the Subread package. All downstream analyses were performed in R v4.5.0 [57]. Gene counts were normalized, and differential expression was conducted using DESeq2 v1.48.0. Data wrangling and visualization were performed with packages from the tidyverse v2.0.0, including ggplot2 v3.5.0 and patchwork v1.3.0 [58,59]. Plots focused on comparing the expression of the transgenic human *SNCA* and endogenous mouse alpha-synuclein (*Snca*).

### 2.7. Exclusion Criteria

#### 2.7.1. Humane Endpoints: Termination Criteria

Animals were monitored for clinical signs as outlined in Supplementary Table S3: Questionnaire for Monitoring Stress in Laboratory Animals. The animals would have been excluded from the experiment and euthanized if it was exhibiting scores greater than 5. None of the mice reached our predefined termination criteria. No significant weight loss was observed during the study, daily variations in body weight did not exceed 2% (approximately ±0.5 g), which is within normal physiological fluctuation. All animals consistently received a score of 0 on the stress monitoring scale.

#### 2.7.2. Exclusion Criteria Based on Technical Difficulties

Seeing that animal behavior was recorded and subsequently analyzed using pre-recorded videos, if the videos used to capture animal behavior were damaged, the animals would have been excluded from analysis. Likewise, exclusion would have occurred if the brain tissue samples for RT-PCR analysis were compromised. None of the mice were excluded based on this criterion.

### 2.8. Statistical Analysis

Statistical analysis was performed using Statistica 12.0 software (StatSoft Inc., Tulsa, OK, USA). Data was graphically expressed as means ± standard deviation (SD), with individual data plots along the column bars. Statistical significance in all statistical comparisons was accepted with *p* ≤ 0.05. The normality of distribution of variables was determined using Shapiro–Wilk’s test. Two-way ANOVA was used to evaluate the interactive effect of GbH and genotype (*housing x genotype*) in regard to the measured parameters of pre-motor PD; post hoc Tukey’s HSD test was applied when appropriate. Student’s *t*-test was used to determine differences between additional C57BL/6J controls and four individual groups when applicable, while if the condition regarding normality of distribution was not satisfied, post hoc Mann–Whitney U test was applied.

## 3. Results

A detailed presentation of the obtained statistical data is available in Tables S1 and S2 in the Supplementary Section.

### 3.1. A53T+ Animals Overexpress SNCA and GbH Had No Impact on SNCA/Snca Expression in A53T+ and A53T− Animals

**Figure 3:**
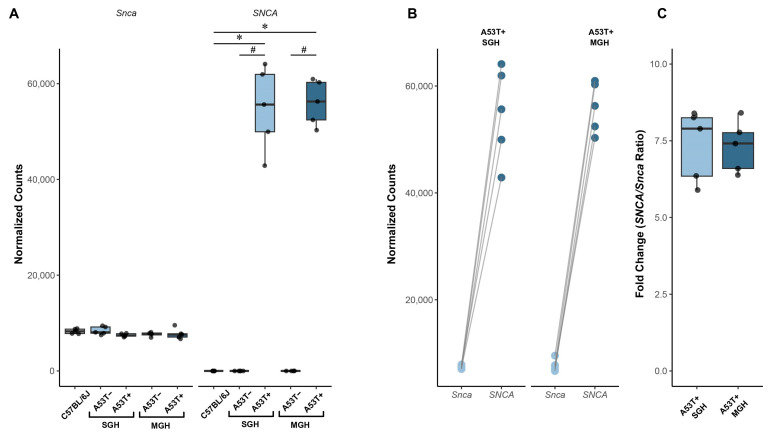
Expression of human *SNCA* transgene and endogenous mouse *Snca* in HPP of adult C57BL/6J, A53T−, and A53T+ male mice. (**A**) Normalized expression counts of human *SNCA* and mouse *Snca* across different groups shown as boxplots with overlaid data points. (**B**) Paired dot plot showing the expression of *SNCA* and *Snca* within individual samples. Points are connected by lines to indicate sample pairing. (**C**) Boxplot representing the fold change between *SNCA* and *Snca* expression (*SNCA*/*Snca* ratio) in group samples, indicating the relative expression level of the inserted transgene compared with the endogenous gene. The data are expressed as mean ± SD, with individual data plots (visible as black dots) along the column bars. Statistical analysis of differential gene expression was performed using DESeq2, applying the Wald tests with Benjamini–Hochberg correction for multiple comparisons. Differences in *SNCA/Snca* expression ratios were evaluated using the Wilcoxon rank-sum test. All tests were two-sided, and a significance threshold of *p* < 0.05 was used (*n* = 5 animals per group). * *p* < 0.05 vs. C57BL/6J group. # vs. A53T−.

Human mutated alpha-synuclein (*SNCA*) was expressed only in the A53T+ mice, without affecting endogenous mouse alpha-synuclein expression (*Snca*; Figure 3A). Predictably, *SNCA* was not detected in the C57BL/6J or A53T– animals (Figure 3A) regardless of their environment. No differences were found between *Snca* expression in all groups. A53T+ expressed *SNCA* at approximately seven times the level of endogenous mouse *Snca* form (Figure 3C) and the environmental conditions had no influence on *SNCA* expression levels in these mice (Figure 3B).

### 3.2. A53T− Mice Kept in SGH as Well as A53T+ Mice Regardless of Housing Conditions Show a Reduced Preference for the Novel Object in Novel Object Recognition Test and Novel Object Location Test

**Figure 4:**
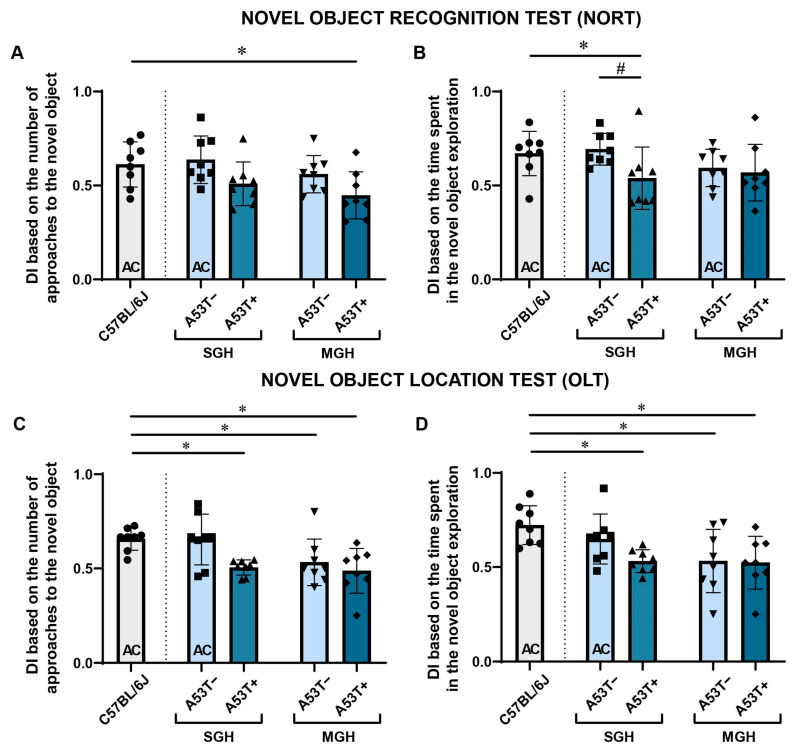
The behavior of adult C57BL/6J, A53T−, and A53T+ male mice in the Novel Object Recognition Test (NORT) and Novel Object Location Test (OLT). The discrimination index (DI) based on the number of approaches (DIna) and exploration time (DIet) in the NORT (**A**,**B**) and in OLT (**C**,**D**) are presented for the C57BL/6J, A53T−, and A53T+ mice kept in SGH and MGH. The data are expressed as mean ± SD, with individual data plots (visible as black symbols) along the column bars (*n* = 8 animals per group). * *p* < 0.05 vs. C57BL/6J group. # vs. A53T−. The AC inside the bars indicates that the DI was significantly above chance (DI > 0.5). The dotted line symbolically separates the bar representing the performance of C57BL/6J mice from the bars representing the performance of JAX006823 strain in a given test.

The discrimination index based on the number of approaches (DIna) indicated an above chance preference for the novel object in C57BL/6J (Figure 4A, one-sample *t*-test: *p* = 0.033, *t* = 2.640) and A53T− mice kept in SGH (Figure 4A, one-sample *t*-test: *p* = 0.019, *t* = 3.051, df = 7) in the NORT. Two-way ANOVA showed a significant effect of *genotype* only, while the post hoc analysis revealed no notable differences in the comparisons of interest.

Compared with C57BL/6J, only A53T+ held in MGH showed lower DIna [Figure 4A, Student’s *t*-test, *p* = 0.018, *t* = −2.685, df = 14]. The discrimination index based on the time spent on novel object exploration (DIet) displayed an above chance preference for the novel object in the C57BL/6J mice (Figure 4B, one-sample *t*-test: *p* = 0.005, *t* = 4.075, df = 7) and the A53T− mice regardless of whether they were being kept in SGH (Figure 4B, one-sample *t*-test: *p* < 0.001, *t* = 6.499, df = 7) or MGH (Figure 4B, one-sample *t*-test: *p* = 0.034, *t* = 2.630, df = 7). Additionally, the statistical analysis revealed that the A53T+ mice kept in SGH display a lower DIet compared with their non-transgenic SGH counterparts [Figure 4B, *U*-test, *p* = 0.018, *Z* = −2.363, *U* = 9] and also compared with the C57BL/6J mice [Figure 4B, *U*-test, *p* = 0.031, *Z* = −2.153, *U* = 11].

Similar changes were observed in both DIna and DIet in the OLT. The one-sample *t*-test showed an above chance preference for the novel object in the C57BL/6J and A53T− mice kept in SGH for both DIna (Figure 4C, *p* < 0.001, *t* = 7.401, df = 7 and Figure 4C, *p* = 0.015, *t* = 3.206, df = 7, respectively) and DIet (Figure 4D, *p* < 0.001, *t* = 6.124, df = 7 and Figure 4D, *p* = 0.015, *t* = 3.193, df = 7, respectively), thus revealing a similar interest in exploring the novel object in both groups. The two-way ANOVA showed only a significant effect of *genotype* regarding DIna, while the post hoc analysis revealed no notable differences in the comparisons of interest. The two-way ANOVA showed no significant differences regarding DIet. The A53T+ mice kept in SGH and the A53T− and A53T+ mice kept in MGH showed lower DIna [Figure 4D, Student’s *t*-test: *p* < 0.001, *t* = −5.909, df = 14; *p* = 0.023, *t* = −2.552, df = 14; *p* = 0.003, *t* = −3.588, df = 14, respectively] and DIet [Figure 4D, Student’s *t*-test: *p* < 0.001, *t* = −4.542, df = 14; *p* = 0.016, *t* = −2.740, df = 14; *p* = 0.006, *t* = −3.245, df = 14, respectively] compared with C57BL/6J.

Additional data regarding NORT and OLT can be found in the Supplementary Section.

### 3.3. The Expression of Iba1, Gfapα, Bdnf, and Il-1β Genes in the HPP Changes in a Genotype-Dependent Manner, Particularly Under MGH Conditions

**Figure 5:**
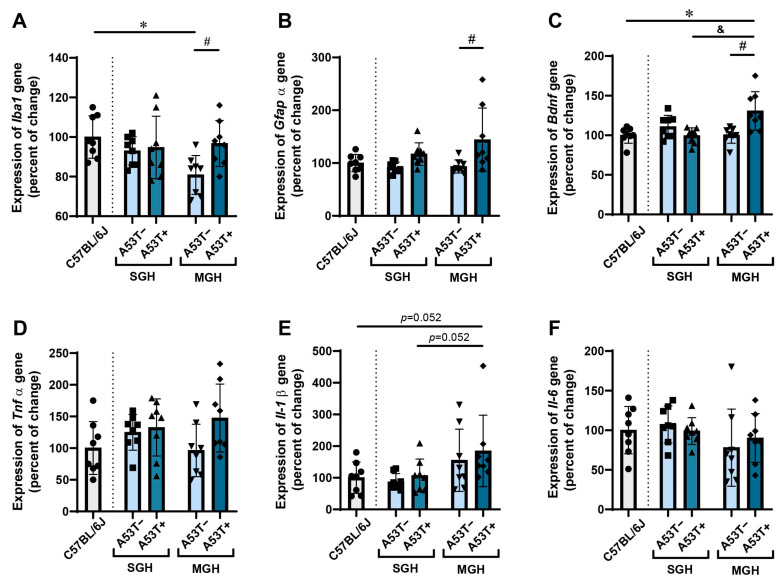
The expression profiles of *Iba1*, *Gfapα*, *Bdnf*, *Tnfα*, *Il-1β*, and *Il-6* genes in the HPP of adult C57BL/6J, A53T−, and A53T+ male mice. Relative expression of *Iba1* (**A**), *Gfapα* (**B**), *Bdnf* (**C**), *Tnfα* (**D**), *Il-1β* (**E**), and *Il-6* (**F**) genes in C57BL6/J, A53T−, and A53T+ mice kept in SGH and MGH are displayed as a percentage of change from C57BL/6J mice. *Gapdh* was used as the reference gene. The data are expressed as mean ± SD, with individual data plots (visible as black symbols) along the column bars (*n* = 8 animals per group). * *p* < 0.05 vs. C57BL/6J group. # vs. A53T−, and & vs. the same genotype in different housing conditions. The dotted line symbolically separates the bars representing the measures obtained from C57BL/6J mice from the bars representing measures obtained from JAX006823 strain.

While the behavioral assessments demonstrated a lack of preference for the novel object in the A53T+ mice regardless of GbH and the A53T− mice maintained in MGH conditions, the gene expression data show a delicate decrease in *Iba1* expression only in the HPP of the A53T− mice maintained in MGH conditions. Specifically, the expression of *Iba1* in the HPP varied in a genotype-dependent manner [Figure 5A, *F*_(1,28)_ = 4.585, *p* = 0.042], with significant differences observed only between the A53T− and A53T+ mice kept in MGH [Figure 5A, Tukey’s *HSD, p* = 0.049]. Additionally, the A53T− mice kept in MGH showed a lower expression of *Iba1* compared with the C57BL/6J controls, also [Figure 5A, Student’s *t*-test: *p* = 0.002, *t* = −3.730, df = 14]. Oppositely, the expression of *Gfapα* was influenced by genotype, and the post hoc analysis revealed a significant increase in *Gfapα* expression in the A53T+ mice kept in MGH compared with their non-transgenic littermates [Figure 5B, Tukey’s *HSD,* # *p* = 0.022]. However, no significant changes were observed between C57BL/6J and all other groups. Similarly, the A53T+ mice kept in MGH showed a significant increase in *Bdnf* expression compared with their non-transgenic littermates [Figure 5D, Tukey’s *HSD,* # *p* = 0.003] and compared with their transgenic counterparts kept in SGH [Figure 5D, Tukey’s *HSD*, and *p* = 0.003]. Additionally, significant differences were observed between the C57BL/6J and A53T+ mice kept in MGH [Figure 5D, Student’s *t*-test: *p* = 0.006, *t* = 3260, df = 14]. There were no significant changes in *Tnfα*, *Il-1β*, and *Il-6* expression in all between-group comparisons of interest (Figure 5D,F).

### 3.4. The Expression of Iba1, Gfapα, Bdnf, and Tnfα in the mPFC Changes in a Genotype- and Housing-Dependent Manner

**Figure 6:**
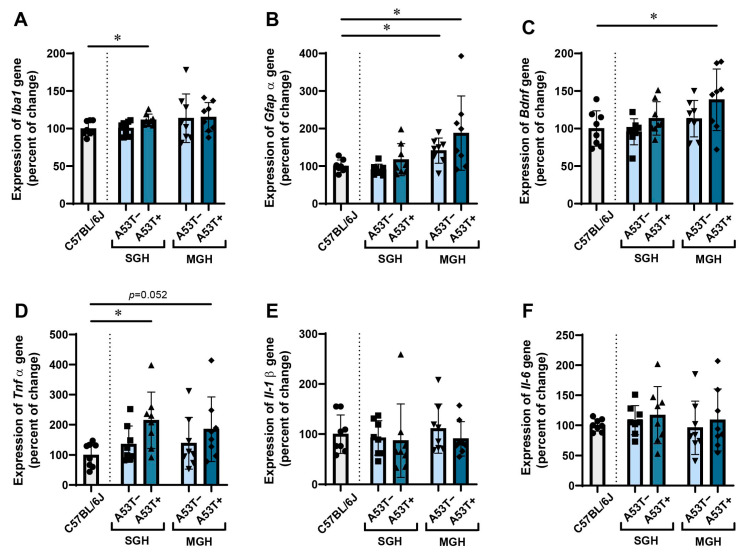
The expression profile of *Iba1*, *Gfapα*, *Bdnf*, *Tnfα*, *Il-1β*, and *Il-6* genes in the mPFC of adult C57BL/6J, A53T−, and A53T+ male mice. Relative expression of *Iba1* (**A**), *Gfapα* (**B**), *Bdnf* (**C**), *Tnfα* (**D**), *Il-1β* (**E**), and *Il-6* (**F**) genes in C57BL/6J mice, A53T−, and A53T+ mice kept in SGH and MGH are displayed as a percentage of change. *Gapdh* was used as the reference gene. The data are expressed as mean ± SD, with individual data plots (visible as black symbols) along the column bars (*n* = 8 animals per group). * *p* < 0.05 vs. C57BL/6J group. The dotted line symbolically separates the bars representing the measures obtained from C57BL/6J mice from the bars representing measures obtained from JAX006823 strain.

While gene expression in the HPP was both different between the A53T− and A53T+ mice and relative to the C57BL/6J controls, the mPFC exhibited the upregulation of inflammatory-related genes and *Bdnf* when compared with those of C57BL/6J controls. Specifically, the expression of *Iba1* in mPFC was not significant in all between-group comparisons, except between the C57BL/6J and A53T+ mice kept in SGH. The two-way ANOVA revealed that the expression of *Gfapα* and *Bdnf* were influenced by housing conditions [Figure 6B,C] and that the expression of *Bdnf* and *Tnf*α changed in a genotype-dependent manner [Figure 6C,D], while post hoc analysis revealed no notable differences in the comparisons of interest. Compared with C57BL/6J, the expression of *Gfapα* increased in both the A53T− and A53T+ mixed housed groups [Figure 6B, Student’s *t*-test: *p* = 0.007, *t* = 3125, df = 14 and Figure 6C, *p* = 0.026, *t* = 2486, df = 14], the expression of *Bdnf* increased in the A53T+ mixed housed group [Figure 6D, Student’s *t*-test: *p* = 0.037, *t* = 2305, df = 14] and the expression of *Tnfα* increased in the A53T+ single housed group [Figure 6D, Student’s *t*-test: *p* = 0.007, *t* = 3.184, df = 14]. There were no significant changes in *Tnfα*, *Il-1β,* and *Il-6* expression in all between-group comparisons of interest (Figure 6E,F).

A comprehensive statistical analysis of gene expression and the corresponding CT values are provided in the Supplementary Section, specifically in Supplementary Tables S1 and S2, respectively.

## 4. Discussion

The RNA-seq data confirmed that *SNCA* was selectively expressed in the A53T+ mice, at levels approximately sevenfold higher than endogenous *Snca* expression, which is in line with the manufacturer’s description of the model (https://www.jax.org/strain/006823 last accessed on 14 June 2025).

Consistent with previous reports, the A53T+ mice displayed significant short-term memory deficits, regardless of housing conditions as they exhibited a lack of preference for the novel object in both the NORT and OLT. These results are in line with studies demonstrating that the A53T mutation leads to early cognitive deficits, particularly in visuo-spatial memory [23] and aligning with the pathophysiology of cognitive symptoms seen in patients with PD [33,60]. The lack of housing-dependent differences in cognitive performance suggests that the A53T mutation exerts a strong effect on memory, independent of the social environment. In contrast, GbH had a significant impact on the A53T− animals, as those housed in MGH performed worse on both visual and spatial memory tasks compared with their counterparts in SGH. In the NORT specifically, the A53T− mice in MGH showed no preference for the novel object based on the DIna, and not Diet, suggesting a change in the appetitive but not in the consummatory aspect of learning as a motivated behavior [61,62]. Appetitive behaviors, representing the more variable and exploratory phase of a behavioral sequence, are often sensitive to external stimuli such as novelty, while consummatory behaviors are more stereotypical and goal directed [63]. This differential impact on exploratory behavior, i.e., the motivational component of learning, points to a possible alteration in the dopaminergic–hippocampal circuitry, which has a key role in exploratory behavior and memory encoding [64]. The effect on spatial memory was even more detrimental as both appetitive and consummatory behaviors were impacted in the OLT. This suggests that exposure to A53T+ animals may negatively influence cognitive performance in non-transgenic littermates potentially through altered social interaction [65] or stress-related factors [66,67]. Although the exact mechanism of the impact of GbH on A53T− mice behavior was beyond the scope of this study, we speculate that the observed changes may be influenced by chronic interaction with A53T+ mice who are reported to have impaired sociability [68]. It should also be noted that although the presence of aggressive traits was not yet officially reported in JAX006823 mice, it is observed in other available A53T lines (e.g., https://www.jax.org/strain/004479 last accessed on 14 June 2025) as well as in the C3H/HeJ strain [69] which was used in the production of the JAX006823 strain (https://www.jax.org/strain/006823 last accessed on 14 June 2025). Furthermore, the potential role of shared gut microbiota, which is known to influence both neuroinflammation and cognitive function [9,70], on the behavior of mice in MGH warrants further investigation in this setting. These insights may have important implications for the design of preclinical research considering that the A53T− mice in SGH exhibited behavioral responses to the novel object that are very similar to those of wild-type C57BL/6J mice while the same was not evident in the MGH conditions.

One important aspect to consider is that most of the following differences in gene expression, especially in the mPFC, are only evident when comparing C57BL/6J and A53T+ animals, which is not in line with the notion that both A53T− and C57BL/6J animals should be treated as equal controls when using the JAX006823 strain. Although there were no apparent differences between the profiles of A53T− in SGH and C57BL/6J animals when considered in isolation, the differences in gene expression were more pronounced when comparing the C57BL/6J mice to the A53T+ mice, emphasizing that researchers should carefully consider control group selection when using this model. Including both groups, when possible, may improve the reliability of experimental results and reduce the variability across different studies using this model.

Our results indicate an increase in the mRNA expression of *Iba1* in the mPFC of the A53T+ animals housed in SGH compared with the C57BL/6J controls, which aligns with previous research on microglial activation in A53T+ animals [23,71], as elevated IBA1 expression suggests heightened microglial reactivity [72]. Interestingly, this was not evident in MGH, highlighting the influence of GbH on the detectability of the parameters of interest in this model. Similarly, *Tnfα* mRNA expression was different in the A53T+ mice regardless of housing conditions compared with the C57BL/6J controls, but in SGH this difference was statistically significant, while in MGH it was a trend (*p* = 0.052) which, again, highlights the influence of GbH on the detectability of relevant parameters in this model. Seeing that no significant changes were detected related to *Il-1β* and *Il-6,* and given that the gene expression of *Iba1* and *Tnfα* in the A53T+ mice was influenced by housing conditions while the behavioral outcomes were not, as well as the fact that the differences were not evident in comparison to A53T−, we speculate that the observed changes in gene expression may not be directly linked to the observed cognitive deficits in the A53T+ mice. Certain authors suggest that TNFα-induced memory consolidation deficits are mediated via the hippocampal expression of IL-1β [73,74], which is also not evident in this study, as hippocampal *Il-1β* was only slightly elevated in the MGH conditions (*p* = 0.052), while cognitive deficits were prevalent in the A53T+ animals regardless of microenvironment.

While there were no differences in *Iba1* expression in the HPP of the A53T+ and A53T− mice, the A53T− animals in MGH exhibited significantly lower *Iba1* levels compared with the C57BL/6J controls. The reduced *Iba1* mRNA expression in the HPP of the A53T− animals in MGH, accompanied by a significant decline in spatial memory performance, is in line with certain studies suggesting a potential association of similar microglial dysfunction (i.e., loss of IBA1 expression) with reduced cognitive function [72]. We speculate that the shared environment could contribute to the cognitive profile of A53T− mice, possibly through effects related to chronic stress [7,8]. Interestingly, when examined in isolation from other groups, it might appear that the A53T+ mice exhibit increased *Iba1* expression compared with the A53T− animals housed in the same social environment, which is in line with previous studies that favor this type of animal housing [23,68]. However, we demonstrated that such observations can be interpreted differently in a wider context. Similarly, the shared environment affected the mRNA expression of *Gfαpα* in the HPP of the A53T+ animals, emphasizing the differences between the A53T+ and A53T− animals in the expression of this marker, which would not be significant otherwise (as seen in SGH). Seeing that an increase in GFAPα in plasma [75] and cerebrospinal fluid [76] is generally associated with cognitive decline in PD, one could easily be inclined to interpret this difference as detrimental when considering the memory impairment observed in A53T+ animals. However, it is important to note that astrocytes have a significant role in maintaining brain homeostasis [77], as they support neuronal survival through mechanisms such as secreting neurotrophins and antioxidants, clearing α-synuclein, and transferring healthy mitochondria to neurons [78]. Furthermore, it is reported that the retroviral transduction of BDNF in astrocytes may ameliorate PD symptoms [78,79]. In the context of this study, considering that the increase was only evident in MGH, we also lean toward an interpretation that favors the protective role of astrocytes and GFAP [80,81] especially since similar differences were observed regarding *Bdnf* expression in A53T+ animals in MGH, and it is known that BDNF can be regulated and produced by astrocytes [82].

BDNF has been widely recognized for its neuroprotective effects, particularly in models of neurodegenerative diseases including PD [35,83]. The observed increase in *Bdnf* expression in the mPFC and the HPP of A53T+ mice could be misinterpreted as a compensatory mechanism aimed at mitigating the early effects of α-synuclein toxicity, as similar findings were reported in other models of PD (e.g., [84]). However, since this increase was not observed in SGH conditions, we suspect that the shared environment could be the factor promoting the expression of *Bdnf* in these animals, since the response was specific to this environmental condition. This interpretation is consistent with findings suggesting that environmental enrichment and social interaction can enhance *Bdnf*/BDNF expression [85,86] although this was not evident on a behavioral level in this model. Nevertheless, again, the differential effects of the GbH on mRNA expression in both A53T+ and A53T− animals emphasize the importance of considering and controlling environmental factors in transgenic animal studies.

The primary aim of this study was to investigate the methodological implications of housing transgenic animals alongside non-transgenic controls. We hope that our work will contribute to the discussion on optimal study designs when using transgenic models. Globally, our data suggests that housing environment, as a confounding variable, may influence behavior, as well as mRNA expression differently in transgenic and non-transgenic animals, with potential implications for future preclinical study designs. One important result of this study reveals the significant impact of GbH on A53T− animals, which are typically used as controls, seeing that the A53T− mice housed in MGH performed worse on both visual and spatial memory tasks compared with their SGH counterparts, who exhibited performance comparable to the C57BL/6J controls. This raises concerns about the reliability of A53T− animals as baseline controls under certain housing conditions. Another interesting point is that A53T+ mice in MGH show elevated *Iba1* expression compared with A53T− mice within the same environment which aligns with the prior literature; however, our data indicates that this apparent increase is highly context-dependent and could lead to misinterpretation. Similarly, *Gfapα* expression in the HPP of A53T+ mice was significantly influenced by the shared environment, resulting in group differences not evident in SGH conditions. Finally, most differences in gene expression were only apparent when comparing C57BL/6J and A53T+ animals, which challenges the assumption that both A53T− and C57BL/6J animals can be used as equal control groups in studies using the JAX006823 strain. The overall conclusion is that the commonly preferred MGH used in preclinical research could influence obtained data and may result in context-dependent assumptions regarding genotype differences.

Although this study is, to the best of our knowledge, the first one to provide data on the interaction between genotype-based environment and genetic predisposition in early PD, it does have several limitations that should be acknowledged. First, the sample consisted of solely male mice, thus limiting the extrapolation of the obtained results to females. The decision to use only male mice in this study was based on clinical data suggesting that males are more susceptible to Parkinson’s disease and related pathologies [87], as well as the fact that female A53T mice have been reported to show less pronounced phenotypes, particularly regarding cognitive deficits and glial activation [88]. Given that our study focused on detecting subtle pre-motor features and associated molecular changes, including female mice who may present with milder or different phenotypes could have introduced additional variability at this exploratory stage. Additionally, while we identified relevant changes on the transcriptional and behavioral level, there is a gap in our understanding of the availability and localization of proteins related to pre-motor features. Immunohistochemistry and Western blot analysis of the expression of cytokines and BDNF can be performed to further explore the effects GbH on parameters related to inflammation and neurotrophic signalization in PD. Another potential limitation of the study concerns the issue of reduced penetrance reported in this model, i.e., that only 90% of hemizygous animals develop overt motor symptoms at 9–16 months of age, and approximately 10% do not exhibit this phenotype (https://www.jax.org/strain/006823 last accessed on 14 June 2025). This phenomenon, where a genotype does not always result in the expected phenotype, is a recognized feature of many inherited diseases [89], and its underlying mechanisms are not understood. While this was out of the scope of our study, which focused on pre-motor features at 6 months of age, well before the onset of motor dysfunction, we disclose that some animals could show variability in later phenotypic expression. However, the data obtained via RNA-seq confirmed *SNCA* overexpression at this age, and no unusual variance in behavioral data within transgenic groups was evident, suggesting a consistent phenotype in the studied cohort. Finaly, GbH undoubtably had an impact on the observed changes in both A53T+ and A53T− mice; however, the underlying mechanisms driving these alterations remain unclear. Future studies should explore specific variables, such as stress, social factors, and microbiota composition in greater detail.

## 5. Conclusions

This study confirmed that the Hualpha-Syn(A53T) transgenic mouse line G2-3 (JAX006823 strain) is a good candidate to model pre-motor short-term visuo-spatial impairment in PD and that GbH can indeed act as a confounding variable in studies using transgenic animals. Overall, our findings imply that non-transgenic littermates housed in SGH conditions represent a more reliable control group than those kept in MGH conditions, at least when considering memory potential and certain biochemical characteristics of the model. Our data also challenges the notion that both A53T− and C57BL/6J animals can be used interchangeably with this strain. The benefits of applying this methodological design in studies investigating other behavioral/molecular features and using different transgenic models remain to be confirmed in future studies. We hope that our work will contribute to the discussion on optimal study designs when using genetically altered mouse lines.

## Figures and Tables

**Table 1:** Primer sequences used in qRT-PCR analysis.

Gene	Forward	Reverse
*Iba1*	5′-GGA TTT GCA GGG AGG AAA AG-3′	5′-TGG GAT CAT CGA GGA ATT G-3′
*Gfapα*	5′-GGA GAT GCG GGA TGG TGA G-3′	5′-ACC ACG TCC TTG TGC TCC TG-3′
*Il-6*	5′-GAC AAA GCC AGA GTC CTT CAG AGA G-3′	5′-CTA GGT TTG CCG AGT AGA TCT C-3′
*Il-1β*	5′-GCA ACT GTT CCT GAA CTC AAC T-3′	5′-ATC TTT TGG GGT CCG TCA ACT-3′
*Tnfα*	5′-CCC TCA CAC TCA GAT CAT CTT CT-3′	5′-GCT ACG ACG TGG GCT ACA G-3′
*Bdnf*	5′-AGG CAC TGG AAC TCG CAA TG-3′	5′-AAG GGC CCG AAC ATA CGA TT-3′
*Gapdh*	5′-TGA AGC AGG CAT CTG AGG G-3′	5′-CGA AGG TGG AAG AGT GGG AG-3′

## Data Availability

The original contributions presented in the study are included in the article/Supplementary Materials. Further inquiries can be directed to the corresponding author.

## Supplementary Materials

The following supporting information can be downloaded at: https://www.mdpi.com/article/10.3390/biomedicines13061506/s1, Figure S1: The behavior of adult C57BL/6J and JAX006823 male mice in first familiarization phase of NORT; Figure S2: Activity directed towards the object exploration C57BL/6J and JAX006823 male mice in the Familiarization phase, Novel Object Recognition Test (NORT) and Novel Object Location Test (OLT). Table S1: The detailed presentation of the obtained statistical data; Table S2: CT values for target and housekeeping genes (mean values from two technical replicates); Table S3: QUESTIONNAIRE FOR MONITORING STRESS IN LABORATORY ANIMALS; Table S4: Ear markings/Mice ID; Table S5: Statistical analysis for supplementary Figure S1; Table S6: Statistical analysis for supplementary Figure S2; Document S1: Ethical approval document.

## Abbreviations

The following abbreviations are used in this manuscript:

A53T+	Hemizygous A53T transgenic mice of the Hualpha-Syn(A53T) transgenic mouse line G2-3 (JAX006823 strain)
A53T−	Non-transgenic littermates of the Hualpha-Syn(A53T) transgenic mouse line G2-3 (JAX006823 strain)
GbH	Genotype-based environment
SGH	Single-genotype environment
MGH	Mixed-genotype environment
PD	Parkinson’s disease
PND	Postnatal day
qPCR	Quantitative polymerase chain reaction
MCI	Mild cognitive impairment
CSF	Cerebrospinal fluid
mRNA	Messenger RiboNucleic Acid
IBA 1	Ionized calcium-binding adaptor molecule 1
GFAPα	Glial Fibrillary Acidic Protein alpha
TNFα	Tumor Necrosis Factor alpha
IL-1β	Interleukin-1 beta
IL-6	Interleukin 6
BDNF	Brain-derived neurotrophic factor
mPFC	Medial prefrontal cortex
HPP	Hippocampus
RT-PCR	Reverse transcription polymerase chain reaction
NORT	Novel Object Recognition Test
OLT	Object Location Test
DI	Discrimination Index
DIna	Discrimination Index based on Number of Approaches
DIet	Discrimination Index based on Exploration Time
GPDH	Glyceraldehyde-3-Phosphate Dehydrogenase
AC	Above chance
SD	Standard deviation
ANOVA	Analysis of variance
