# Correction: Dubljević et al. Genotype-Based Housing as a Potential Confounder in Studies Using Transgenic Mouse Models—Insight from the A53T Mouse Model of Parkinson’s Disease. *Biomedicines* 2025, *13*, 1506

**DOI:** 10.3390/biomedicines13123094

**Published:** 2025-12-16

**Authors:** Olga Dubljević, Miodrag Dragoj, Milica Potrebić Stefanović, Maja Srbovan, Miloš Stanojlović, Željko Pavković

**Affiliations:** 1 Department of Neurobiology, Institute for Biological Research “Siniša Stanković”—National Institute of Republic of Serbia, University of Belgrade, 11060 Belgrade, Serbia; miodrag.dragoj@ibiss.bg.ac.rs (M.D.); milica.potrebic@ibiss.bg.ac.rs (M.P.S.); maja.srbovan@ibiss.bg.ac.rs (M.S.); milos.stanojlovic@ibiss.bg.ac.rs (M.S.); zeljko.pavkovic@ibiss.bg.ac.rs (Ž.P.); 2 Department of Pharmacology, Toxicology and Pharmacy, University of Veterinary Medicine Hannover, Foundation, 30559 Hannover, Germany

## Acknowledgments Correction

There was an error in the original publication [1]. The Acknowledgments Section is missing a sentence.

A correction has been made to the Acknowledgments Section:

The results presented in this manuscript are in line with Sustainable Development Goal 3 (Good Health and Well-being) of the United Nations 2030 Agenda. We thank Vesna Pešić for her valuable contributions to the research design of the study, as well as advice, overview, and suggestions regarding the behavioral paradigms applied in this study. Additionally, we thank Franziska Richter Asenzio (Department of Pharmacology, Toxicology and Pharmacy, University of Veterinary Medicine, Hannover, Germany) for providing us with the breeding mice of the JAX006823 strain.

The authors state that the scientific conclusions are unaffected. This correction was approved by the Academic Editor. The original publication has also been updated.
