# Supplementary material for: Genotype-Based Housing as a Potential Confounder in Studies Using Transgenic Mouse Models—Insight from the A53T Mouse Model of Parkinson’s Disease
Source: Biomedicines. 2025 Jun 19;13(6):1506. doi: 10.3390/biomedicines13061506 (PMC12190588; doi:10.3390/biomedicines13061506)
Supplement: Supplementary file 1 [file biomedicines-13-01506-s001.zip › biomedicines-3607422-supplementary.pdf]

Supplementary material

Supplementary table S1

Figure 4A

Test	Post hoc	Group	Comparison	p
**Two-way ANOVA:**<br>Housing: F (1, 28) =2.787, p = 0.106<br>Genotype: F (1, 28) =8.421, p = 0.007<br>Housing x Genotype: F (1, 28) =0.347, p = 0.853	**Post hoc: Tuckey HSD**	SGH	A53T- vs. A53T+	p = 1.152
		MGH	A53T- vs. A53T+	p = 0.242
		A53T-	MGH vs. SGH	p = 0.563
		A53T+	MGH vs. SGH	p = 0.722

Test		Group	t	df	p
one sample t-test	C57BL/6J		2.640	7	0.033
	SGH	A53T -	0.219	7	0.019
		A53T +	3.051	7	0.833
	MGH	A53T -	1.199	7	0.130
		A53T +	1.714	7	0.269

Test			Group	t	df	p
Student’s t-test	C57BL/ 6J vs.	SGH	A53T -	0.402	14	0.693
			A53T +	-1.747	14	0.102
		MGH	A53T -	-0.948	14	0.359
			A53T +	-2.685	14	0.018

Figure 4B

Test	Group	Comparison	U	Z	p
Mann Whitney U test	SGH	A53T- vs. A53T+	9	-2.363	0.018
	MGH	A53T- vs. A53T+	26.5	-0.525	0.600
	A53T-	MGH vs. SGH	29	-0.263	0.793
	A53T+	MGH vs. SGH	16	1.628	0.104

Test		Group	t	df	p
one sample t-test	C57BL/6J		4.075	7	0.005
	SGH	A53T -	6.499	7	<0.001
		A53T +	0.665	7	0.528
	MGH	A53T -	2.630	7	0.034
		A53T +	1.282	7	0.241

Test			Group	U	Z	p
Mann Whitney U test	C57BL/ 6J vs.	SGH	A53T -	32	14	1.000
			A53T +	11	14	0.031
		MGH	A53T -	18.5	14	0.172
			A53T +	17	14	0.127

Figure 4C

Test	Post hoc	Group	Comparison	p
**Two-way ANOVA:**<br>Housing: F (1, 28) =3.107, p = 0.089<br>Genotype: F (1, 28) =6.082, p = 0.020<br>Housing x Genotype: F (1, 28) =1.710, p = 0.202	**Post hoc: Tuckey HSD**	SGH	A53T- vs. A53T+	p = 0.057
		MGH	A53T- vs. A53T+	p = 0.845
		A53T-	MGH vs. SGH	p = 0.156
		A53T+	MGH vs. SGH	p = 0.988

Test		Group	t	df	p
one sample t-test	C57BL/6J		7.401	7	<0.001
	SGH	A53T -	3.206	7	0.015
		A53T +	0.345	7	0.740
	MGH	A53T -	0.753	7	0.476
		A53T +	0.306	7	0.768

Test			Group	t	df	p
Student’s t-test	C57BL/ 6J vs.	SGH	A53T -	-0.069	14	0.946
			A53T +	-5.909	14	<0.001
		MGH	A53T -	-2.552	14	0.023
			A53T +	-3.588	14	0.003

Supplementary material

Figure 4D

Two-way ANOVA:	Post hoc analysis:
Housing: F (1, 28) =1.814, p = 0.188<br>Genotype: F (1, 28) =1.831, p = 0.187<br>Housing x Genotype: F (1, 28) =1.372, p = 0.251	Not applicable

			t	df	p
one sample t-test		C57BL/6J	6.124	7	<0.001
	SGH	A53T -	3.193	7	0.015
		A53T +	1.509	7	0.175
	MGH	A53T -	0.539	7	0.607
		A53T +	0.465	7	0.656

				t	df	p
Student's t-test	C57BL/ 6J vs.	SGH	A53T -	-1.251	14	0.231
			A53T +	-4.542	14	<0.001
		MGH	A53T -	-2.740	14	0.016
			A53T +	-3.245	14	0.006

Figure 5A

Two-way ANOVA:	Post hoc: Tuckey HSD			
Housing: F (1, 28) =1.620, p = 0.213<br>Genotype: F (1, 28) =4.585, p = 0.041<br>Housing x Genotype: F (1, 28) =2.992, p = 0.095		SGH	A53T- vs. A53T+	p = 0.991
		MGH	A53T- vs. A53T+	p = 0.049
		A53T-	MGH vs. SGH	p = 0.171
		A53T+	MGH vs. SGH	p = 0.988

				t	df	p
Student's t-test	C57BL/ 6J vs.	SGH	A53T -	-1.534	14	0.147
			A53T +	-0.787	14	0.445
		MGH	A53T -	-3.730	14	0.002
			A53T +	-0.616	14	0.548

Figure 5B

Two-way ANOVA:	Post hoc: Tuckey HSD			
Housing: F (1, 28) =6.098, p = 0.216<br>Genotype: F (1, 28) =5.198, p = 0.002<br>Housing x Genotype: F (1, 28) =5.198, p = 0.288		SGH	A53T- vs. A53T+	p = 0.418
		MGH	A53T- vs. A53T+	p = 0.022
		A53T-	MGH vs. SGH	p = 0.999
		A53T+	MGH vs. SGH	p = 0.363

				t	df	p
Student's t-test	C57BL/ 6J vs.	SGH	A53T -	-1.256	14	0.230
			A53T +	-1.255	14	0.230
		MGH	A53T -	-0.892	14	0.387
			A53T +	2.012	14	0.064

Figure 5C

Two-way ANOVA:	Post hoc: Tuckey HSD			
Housing: F (1, 28) =3.198, p = 0.084<br>Genotype: F (1, 28) =3.047, p = 0.092<br>Housing x Genotype: F (1, 28) =13.836, p<0.001		SGH	A53T- vs. A53T+	p = 0.512
		MGH	A53T- vs. A53T+	p = 0.003
		A53T-	MGH vs. SGH	p = 0.531
		A53T+	MGH vs. SGH	p = 0.003

				t	df	p
Student's t-test	C57BL/ 6J vs.	SGH	A53T -	1.738	14	0.104
			A53T +	-0.047	14	0.963
		MGH	A53T -	0.000	14	1.000
			A53T +	3.260	14	0.006

Supplementary material

Figure 5D

Two-way ANOVA	Post hoc
**Two-way ANOVA:**<br>Housing: F (1, 28) =0.202, p = 0.656<br>Genotype: F (1, 28) =3.765, p = 0.062<br>Housing x Genotype: F (1, 28) =2.071, p = 0.161	post hoc analysis<br>not applicable

Test	Comparison	Housing	Genotype	t	df	p
Student's t-test	C57BL/6J vs.	SGH	A53T -	1.407	14	0.181
			A53T +	1.502	14	0.155
		MGH	A53T -	-0.181	14	0.859
			A53T +	1.983	14	0.067

Figure 5E

Test	Group	Comparison	U	Z	p
Mann Whitney U test	SGH	A53T- vs. A53T+	72	-0.368	0.713
	MGH	A53T- vs. A53T+	77	-0.893	0.372
	A53T-	MGH vs. SGH	82	-1.418	0.156
	A53T+	MGH vs. SGH	87	-1.943	0.052

Test	Comparison	Housing	Genotype	U	Z	p
Mann Whitney U test	C57BL/6J vs.	SGH	A53T -	29	-0.263	0.793
			A53T +	28	0.368	0.713
		MGH	A53T -	22	0.998	0.318
			A53T +	13	1.943	0.052

Figure 5F

Two-way ANOVA	Post hoc
**Two-way ANOVA:**<br>Housing: F (1, 28) =2.827, p = 0.104<br>Genotype: F (1, 28) =0.024, p = 0.879<br>Housing x Genotype: F (1, 28) = 0.815, p = 0.374	post hoc analysis<br>not applicable

Test	Comparison	Housing	Genotype	t	df	p
Student's t-test	C57BL/6J vs.	SGH	A53T -	0.557	14	0.586
			A53T +	-0.086	14	0.933
		MGH	A53T -	-1.087	14	0.295
			A53T +	-0.654	14	0.524

Figure 6A

Two-way ANOVA	Post hoc
**Two-way ANOVA:**<br>Housing: F (1, 28) =1.430, p = 0.241<br>Genotype: F (1, 28) =0.850, p = 0.364<br>Housing x Genotype: F (1, 28) =0.445, p = 0.510	post hoc analysis<br>not applicable

Test	Comparison	Housing	Genotype	t	df	p
Student's t-test	C57BL/6J vs.	SGH	A53T -	0.123	14	0.904
			A53T +	2.732	14	0.016
		MGH	A53T -	1.141	14	0.273
			A53T +	2.002	14	0.065

Figure 6B

Two-way ANOVA	Post hoc	Group	Comparison	p
**Two-way ANOVA:**<br>Housing: F (1, 28) =9.098, p = 0.005<br>Genotype: F (1, 28) =3.331, p = 0.079<br>Housing x Genotype: F (1, 28) =0.251, p = 0.620	**Post hoc: Tuckey HSD**	SGH	A53T- vs. A53T+	p = 0.786
		MGH	A53T- vs. A53T+	p = 0.371
		A53T-	MGH vs. SGH	p = 0.304
		A53T+	MGH vs. SGH	p = 0.084

Supplementary material

Test		Housing	Group	t	df	p
Student's t-test	C57BL/6J vs.	SGH	A53T -	-1.217	14	0.244
			A53T +	1.068	14	0.304
		MGH	A53T -	3.125	14	0.007
			A53T +	2.486	14	0.026

Figure 6C

Two-way ANOVA	Post hoc: Tuckey HSD			
**Two-way ANOVA:**<br>Housing: F (1, 28) =4.635, p = 0.040<br>Genotype: F (1, 28) =4.828, p = 0.036<br>Housing x Genotype: F (1, 28) =0.159, p = 0.693		SGH	A53T- vs. A53T+	p = 0.588
		MGH	A53T- vs. A53T+	p = 0.279
		A53T-	MGH vs. SGH	p = 0.607
		A53T+	MGH vs. SGH	p = 0.293

Test		Housing	Group	t	df	p
Student's t-test	C57BL/6J vs.	SGH	A53T -	-0.397	14	0.697
			A53T +	1.181	14	0.257
		MGH	A53T -	1.091	14	0.293
			A53T +	2.305	14	0.037

Figure 6D

Two-way ANOVA	
**Two-way ANOVA:**<br>Housing: F (1, 28) =0.179, p = 0.675<br>Genotype: F (1, 28) =4.041, p = 0.054<br>Housing x Genotype: F (1, 28) =0.270, p = 0.607	post hoc analysis not applicable

Test		Housing	Group	t	df	p
Student's t-test	C57BL/6J vs.	SGH	A53T -	1.396	14	0.185
			A53T +	3.184	14	0.007
		MGH	A53T -	1.154	14	0.268
			A53T +	2.109	14	0.052

Figure 6E

Test			U	Z	p
Mann Whitney U test	SGH	A53T- vs. A53T+	23	0.893	0.372
	MGH	A53T- vs. A53T+	25	0.683	0.495
	A53T-	MGH vs. SGH	26	-0.578	0.564
	A53T+	MGH vs. SGH	20	-1.208	0.227

Test		Housing	Group	U	Z	p
Mann Whitney U test	C57BL/6J vs.	SGH	A53T -	28	-0.368	0.713
			A53T +	19	-1.313	0.189
		MGH	A53T -	28	0.368	0.713
			A53T +	31	-0.053	0.958

Figure 6F

Two-way ANOVA	
**Two-way ANOVA:**<br>Housing: F (1, 28) =0.446, p = 0.510<br>Genotype: F (1, 28) =0.490, p = 0.490<br>Housing x Genotype: F (1, 28) =0.019, p = 0.891	post hoc analysis not applicable

Test		Housing	Group	t	df	p
Student's t-test	C57BL/6J vs.	SGH	A53T -	0.928	14	0.369
			A53T +	1.000	14	0.334
		MGH	A53T -	-0.232	14	0.820
			A53T +	0.489	14	0.633

Supplementary material

Supplementary table S2. CT values for target and housekeeping genes (mean values from two technical replicates)										
PREFRONTAL CORTEX										
biological sample	C57BL/6J		A53T- (SGH)		A53T+ (SGH)		A53T- (MGH)		A53T+ (MGH)	
	Iba1	*Gapdh*	*Iba1*	*Gapdh*	*Iba1*	*Gapdh*	*Iba1*	*Gapdh*	*Iba1*	*Gapdh*
1	23.990	15.470	24.335	15.715	23.995	15.874	23.590	15.245	23.694	15.700
2	24.063	15.766	24.379	15.975	24.127	15.809	23.385	15.377	23.280	15.166
3	24.005	15.472	24.285	15.890	23.969	15.581	23.555	14.932	23.603	15.454
4	24.103	15.792	24.335	15.978	23.573	15.374	23.802	15.053	23.373	15.424
5	23.946	15.471	23.879	15.585	24.929	16.660	23.501	15.883	23.728	15.094
6	24.025	15.725	23.923	15.599	23.960	15.584	23.832	15.404	23.411	15.083
7	24.499	15.824	24.174	15.622	23.996	15.687	23.263	15.164	23.628	15.133
8	24.220	15.686	24.354	15.725	24.004	15.524	23.883	15.256	23.420	14.990
biological sample	C57BL/6J		A53T- (SGH)		A53T+ (SGH)		A53T- (MGH)		A53T+ (MGH)	
	Gfap α	*Gapdh*	*Gfap α*	*Gapdh*	*Gfap α*	*Gapdh*	*Gfap α*	*Gapdh*	*Gfap α*	*Gapdh*
1	22.183	15.470	22.327	15.715	22.785	15.874	21.574	15.245	21.886	15.700
2	21.949	15.766	22.564	15.975	21.951	15.809	21.266	15.377	20.610	15.166
3	22.127	15.472	22.674	15.890	21.444	15.581	20.931	14.932	22.127	15.454
4	22.408	15.792	22.253	15.978	20.933	15.374	21.920	15.053	19.994	15.424
5	22.384	15.471	22.218	15.585	23.441	16.660	21.706	15.883	21.649	15.094
6	22.244	15.725	22.435	15.599	22.340	15.584	21.022	15.404	20.375	15.083
7	22.139	15.824	22.545	15.622	22.189	15.687	21.127	15.164	21.184	15.133
8	22.249	15.686	22.680	15.725	22.125	15.524	21.467	15.256	20.541	14.990
biological sample	C57BL/6J		A53T- (SGH)		A53T+ (SGH)		A53T- (MGH)		A53T+ (MGH)	
	Bdnf	*Gapdh*	*Bdnf*	*Gapdh*	*Bdnf*	*Gapdh*	*Bdnf*	*Gapdh*	*Bdnf*	*Gapdh*
1	22.918	15.470	22.482	15.715	22.907	15.874	22.166	15.245	22.152	15.700
2	22.738	15.766	23.770	15.975	22.608	15.809	22.136	15.377	21.299	15.166
3	22.821	15.472	22.969	15.890	22.550	15.581	22.285	14.932	22.973	15.454
4	22.987	15.792	23.122	15.978	21.936	15.374	22.013	15.053	21.898	15.424
5	22.973	15.471	22.580	15.585	23.120	16.660	22.349	15.883	21.611	15.094
6	22.530	15.725	22.814	15.599	22.875	15.584	22.031	15.404	22.098	15.083
7	22.398	15.824	22.699	15.622	22.743	15.687	22.545	15.164	22.046	15.133
8	22.542	15.686	22.758	15.725	22.503	15.524	22.043	15.256	21.138	14.990
biological sample	C57BL/6J		A53T- (SGH)		A53T+ (SGH)		A53T- (MGH)		A53T+ (MGH)	
	Tnfα	*Gapdh*	*Tnfα*	*Gapdh*	*Tnfα*	*Gapdh*	*Tnfα*	*Gapdh*	*Tnfα*	*Gapdh*
1	31.792	15.470	30.526	15.715	30.025	15.874	34.536	17.488	32.743	17.585
2	31.522	15.766	31.811	15.975	31.676	15.809	33.426	17.202	31.329	17.236
3	32.186	15.472	32.314	15.890	30.352	15.581	33.825	17.234	32.350	17.518
4	31.525	15.792	32.178	15.978	30.465	15.374	33.381	17.376	33.153	17.354
5	31.065	15.471	31.163	15.585	32.011	16.660	32.494	17.457	33.452	17.262
6	31.502	15.725	30.778	15.599	30.492	15.584	32.024	17.521	33.399	17.294
7	32.697	15.824	31.779	15.622	30.631	15.687	32.769	17.175	33.506	17.010
8	33.002	15.686	32.063	15.725	31.782	15.524	33.399	17.294	32.434	17.112
biological sample	C57BL/6J		A53T- (SGH)		A53T+ (SGH)		A53T- (MGH)		A53T+ (MGH)	
	Il-1β	*Gapdh*	*Il-1β*	*Gapdh*	*Il-1β*	*Gapdh*	*Il-1β*	*Gapdh*	*Il-1β*	*Gapdh*
1	28.640	15.470	30.206	15.715	30.459	15.874	28.794	15.245	29.879	15.700
2	29.441	15.766	29.945	15.975	29.978	15.809	28.996	15.377	28.313	15.166
3	29.867	15.472	29.777	15.890	30.898	15.581	29.200	14.932	29.851	15.454
4	30.378	15.792	30.460	15.978	30.755	15.374	29.412	15.053	28.864	15.424
5	29.193	15.471	30.518	15.585	31.087	16.660	29.068	15.883	29.170	15.094
6	29.921	15.725	29.014	15.599	30.001	15.584	28.146	15.404	29.752	15.083

Supplementary material

7	28.992	15.824	29.169	15.622	28.114	15.687	29.195	15.164	29.210	15.133
8	29.879	15.686	29.069	15.725	29.271	15.524	29.540	15.256	29.145	14.990
biological sample	C57BL/6J		A53T- (SGH)		A53T+ (SGH)		A53T- (MGH)		A53T+ (MGH)	
	Il-6	*Gapdh*	*Il-6*	*Gapdh*	*Il-6*	*Gapdh*	*Il-6*	*Gapdh*	*Il-6*	*Gapdh*
1	30.939	15.470	31.146	15.715	30.526	15.874	31.158	15.245	30.701	15.700
2	31.452	15.766	31.566	15.975	31.008	15.809	31.559	15.377	31.075	15.166
3	31.041	15.472	31.778	15.890	30.693	15.581	31.899	14.932	30.812	15.454
4	31.658	15.792	31.283	15.978	30.992	15.374	31.145	15.053	31.942	15.424
5	31.142	15.471	30.659	15.585	32.719	16.660	31.515	15.883	30.848	15.094
6	31.456	15.725	31.234	15.599	32.172	15.584	30.182	15.404	31.032	15.083
7	31.677	15.824	31.736	15.622	31.583	15.687	30.440	15.164	29.749	15.133
8	31.221	15.686	31.320	15.725	30.786	15.524	31.203	15.256	31.241	14.990
HIPPOCAMPUS										
biological sample	C57BL/6J		A53T- (SGH)		A53T+ (SGH)		A53T- (MGH)		A53T+ (MGH)	
	Iba1	*Gapdh*	*Iba1*	*Gapdh*	*Iba1*	*Gapdh*	*Iba1*	*Gapdh*	*Iba1*	*Gapdh*
1	24.421	15.812	24.297	15.689	24.334	15.602	24.451	15.380	23.430	15.027
2	24.001	15.692	24.195	15.630	24.300	15.438	24.089	15.349	23.720	15.145
3	24.138	15.592	24.229	15.505	24.214	15.617	24.220	15.189	24.867	15.705
4	24.129	15.769	23.845	15.369	23.987	15.273	23.480	14.780	23.775	14.990
5	24.135	15.758	23.954	15.437	24.267	15.954	23.600	15.029	24.273	15.734
6	23.688	14.974	24.038	15.302	24.863	16.629	24.394	15.410	23.844	15.127
7	24.003	15.455	23.926	15.384	24.180	15.625	24.398	15.638	23.255	14.740
8	24.188	15.514	24.140	15.367	24.333	15.503	23.893	15.139	27.226	18.927
biological sample	C57BL/6J		A53T- (SGH)		A53T+ (SGH)		A53T- (MGH)		A53T+ (MGH)	
	Gfap α	*Gapdh*	*Gfap α*	*Gapdh*	*Gfap α*	*Gapdh*	*Gfap α*	*Gapdh*	*Gfap α*	*Gapdh*
1	20.007	15.812	20.013	15.689	19.650	15.602	19.926	15.549	19.812	15.408
2	19.570	15.692	19.875	15.630	19.827	15.438	19.468	15.512	19.246	15.246
3	19.996	15.592	19.901	15.505	19.136	15.617	19.703	15.347	20.032	15.851
4	19.815	15.769	19.810	15.369	19.186	15.273	19.367	15.138	18.863	15.250
5	19.808	15.758	19.594	15.437	19.853	15.954	19.709	15.289	19.651	15.703
6	19.618	14.974	19.914	15.302	20.598	16.629	19.986	15.483	18.248	15.408
7	19.816	15.455	19.826	15.384	19.788	15.625	20.206	15.740	19.172	15.096
8	19.730	15.514	19.500	15.367	19.607	15.503	19.580	15.382	21.412	18.280
biological sample	C57BL/6J		A53T- (SGH)		A53T+ (SGH)		A53T- (MGH)		A53T+ (MGH)	
	Bdnf	*Gapdh*	*Bdnf*	*Gapdh*	*Bdnf*	*Gapdh*	*Bdnf*	*Gapdh*	*Bdnf*	*Gapdh*
1	22.249	15.130	22.316	15.812	22.390	15.689	22.249	15.130	21.340	14.904
2	21.980	15.247	22.190	15.692	22.336	15.630	21.980	15.247	21.665	15.460
3	22.035	15.396	22.468	15.592	22.131	15.505	22.035	15.396	21.860	15.358
4	22.274	15.470	22.263	15.769	22.062	15.369	22.274	15.470	22.116	15.656
5	22.424	15.755	22.090	15.758	22.342	15.437	22.424	15.755	21.500	15.326
6	21.923	15.324	21.742	14.974	22.339	15.302	21.923	15.324	21.116	15.170
7	22.176	15.410	22.137	15.455	21.995	15.384	22.176	15.410	21.627	14.939
8	21.972	15.216	22.281	15.514	22.190	15.367	21.972	15.216	24.496	17.796
biological sample	C57BL/6J		A53T- (SGH)		A53T+ (SGH)		A53T- (MGH)		A53T+ (MGH)	
	Tnfα	*Gapdh*	*Tnfα*	*Gapdh*	*Tnfα*	*Gapdh*	*Tnfα*	*Gapdh*	*Tnfα*	*Gapdh*
1	31.403	15.812	30.850	15.689	30.837	15.602	30.957	15.130	29.971	14.904
2	31.407	15.692	30.895	15.630	31.664	15.438	31.861	15.247	31.146	15.460
3	31.835	15.592	31.864	15.505	31.174	15.617	30.467	15.396	31.071	15.358
4	30.792	15.769	31.028	15.369	30.530	15.273	31.959	15.470	30.416	15.656
5	31.143	15.758	30.806	15.437	30.990	15.954	31.101	15.755	31.062	15.326
6	31.282	14.974	30.771	15.302	31.615	16.629	32.156	15.324	31.220	15.170
7	31.844	15.455	31.083	15.384	30.788	15.625	31.190	15.410	29.544	14.939
8	32.349	15.514	30.727	15.367	32.169	15.503	31.234	15.216	31.651	17.796

Supplementary material

biological sample	C57BL/6J		A53T- (SGH)		A53T+ (SGH)		A53T- (MGH)		A53T+ (MGH)	
	Il-1β	*Gapdh*	*Il-1β*	*Gapdh*	*Il-1β*	*Gapdh*	*Il-1β*	*Gapdh*	*Il-1β*	*Gapdh*
1	29.781	15.812	31.010	15.689	30.415	15.602	31.645	16.886	30.849	16.488
2	30.430	15.692	30.958	15.630	31.170	15.438	29.690	16.599	30.314	16.552
3	31.124	15.592	29.995	15.505	31.052	15.617	31.023	16.958	31.510	16.951
4	31.800	15.769	30.365	15.369	30.801	15.273	29.927	16.585	29.424	16.790
5	30.013	15.758	30.790	15.437	30.619	15.954	32.068	16.703	31.299	16.517
6	31.044	14.974	30.349	15.302	31.335	16.629	31.901	16.410	30.635	16.464
7	30.218	15.455	29.833	15.384	28.187	15.625	30.939	16.511	30.600	16.251
8	30.082	15.514	30.920	15.367	29.257	15.503	31.743	16.943	33.194	19.557
biological sample	C57BL/6J		A53T- (SGH)		A53T+ (SGH)		A53T- (MGH)		A53T+ (MGH)	
	Il-6	*Gapdh*	*Il-6*	*Gapdh*	*Il-6*	*Gapdh*	*Il-6*	*Gapdh*	*Il-6*	*Gapdh*
1	30.871	15.812	31.803	15.689	30.763	15.602	33.534	16.886	31.783	16.488
2	31.317	15.692	30.716	15.630	30.945	15.438	32.673	16.599	33.337	16.552
3	31.609	15.592	30.971	15.505	31.179	15.617	32.928	16.958	32.727	16.951
4	31.062	15.769	30.544	15.369	30.955	15.273	33.564	16.585	33.155	16.790
5	31.552	15.758	30.927	15.437	31.389	15.954	33.773	16.703	32.163	16.517
6	30.408	14.974	31.086	15.302	32.234	16.629	31.748	16.410	32.252	16.464
7	31.978	15.455	30.893	15.384	31.350	15.625	31.216	16.511	31.344	16.251
8	30.723	15.514	30.606	15.367	31.531	15.503	33.140	16.943	35.126	19.557

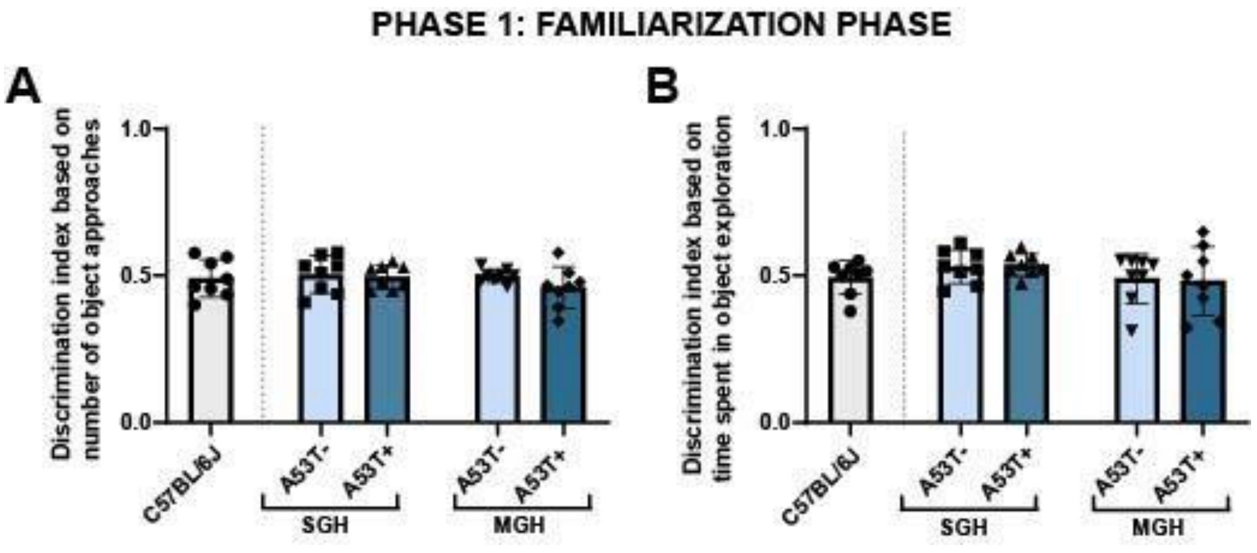

Supplementary material

Supplementary figure S1. The behavior of adult C57BL/6J and JAX006823 male mice in first familiarization phase of NORT. The discrimination indexes based on the number of approaches (A) and exploration time (B) and in the first familiarization phase are presented for the C57BL/6J, A53T- and A53T+ mice kept in single genotype housing (SHG) and mixed genotype housing (MSH). The data are expressed as mean ± SD, with individual data plots along the column bars (n = 8 animals per group).

Table S5. Statistical analysis for supplementary Figure S1						
**Figure 1A**						
**Two-way ANOVA:**<br>Housing: F (1, 28) =1.210, p = 0.281<br>Genotype: F (1, 28) =1.676, p = 0.206<br>Housing x Genotype: F (1, 28) =0.695, p = 0.411	Post hoc test<br>Not applicable					
				**t**	**df**	**p**
Student’s t-test	C57BL/6 J vs.	SGH	A53T -	0.439	14	0.847
			A53T +	0.197	14	0.667
		MGH	A53T -	0.370	14	0.717
			A53T +	-0.911	14	0.377
**Figure 1B**						
				**U**	**Z**	**p**
Mann Whitney U test		SGH	A53T- vs. A53T+	30	1.157	0.875
		MGH	A53T- vs. A53T+	31	-0.053	0.958
		A53T-	MGH vs. SGH	22	0.788	0.431
		A53T+	MGH vs. SGH	24	0.998	0.318
				**U**	**Z**	**p**
Mann Whitney U test	C57BL/6 J vs.	SGH	A53T -	20	1.208	0.228
			A53T +	17	1.523	0.128
		MGH	A53T -	29	0.263	0.793
			A53T +	28.5	-0.315	0.753

Supplementary material

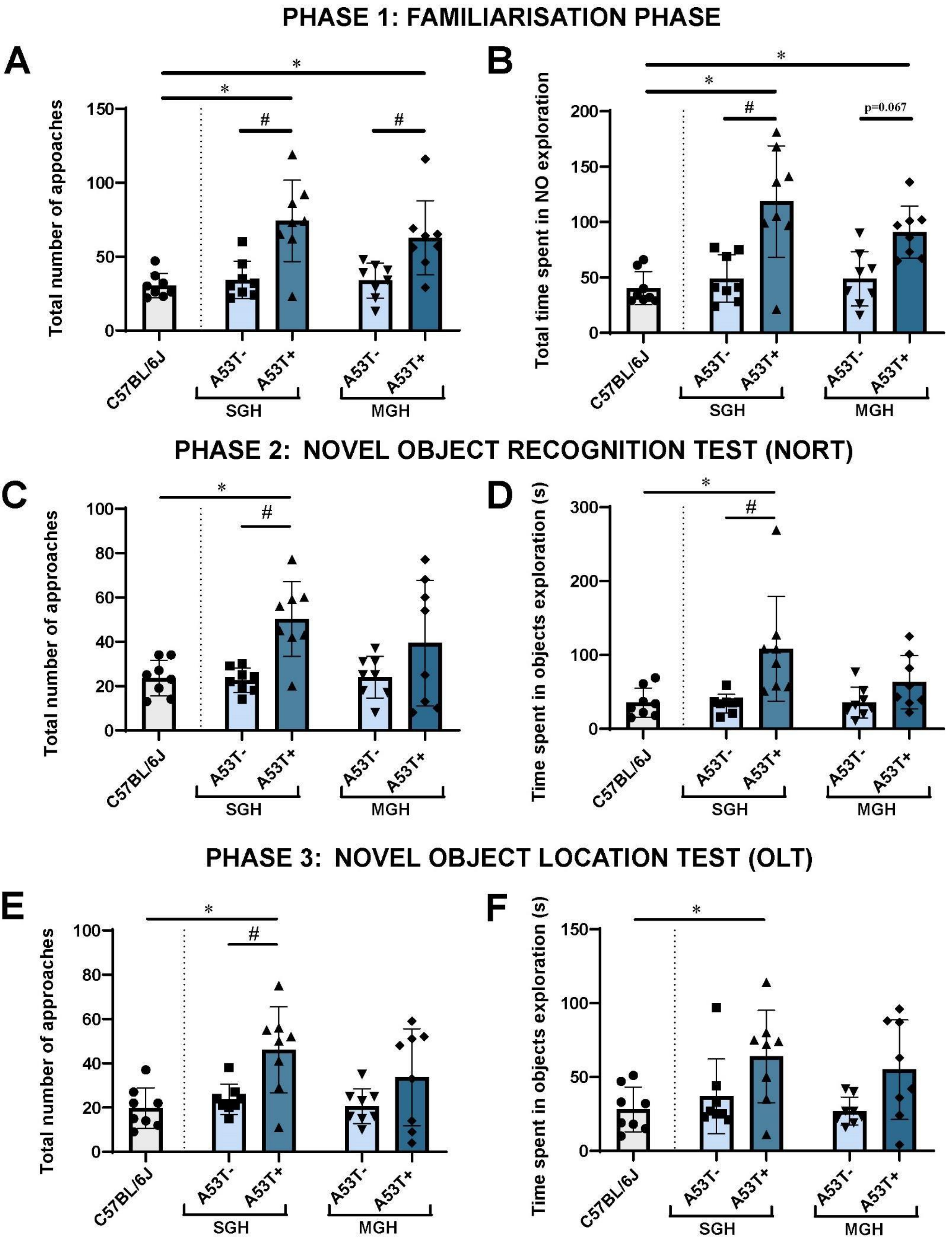

Supplementary material

Supplementary figure S2. Activity directed towards the object exploration C57BL/6J and JAX006823 male mice in the Familiarization phase, Novel Object Recognition Test (NORT) and Novel Object Location Test (OLT). The total number of approaches to the objects and total time spent in object exploration and in the Familiarization phase (A, B), in the Novel Object Recognition Test (C, D) and in the Novel Object Relocation Test (E, F) are presented for the C57BL/6J, A53T- and A53T+ mice kept in single genotype housing (SHG) and mixed genotype housing (MSH). The data are ex-pressed as mean ± SD, with individual data plots along the column bars (n = 8 animals per group). *p < 0.05 vs C57BL6/j group. # vs. A53T-.

Table S6. Statistical analysis for supplementary Figure S2

Figure 2A

**Two-way ANOVA:** Housing: F (1, 28) = 0.683, p = 0.415; Genotype: F (1, 28) =22.586, p <0.001; Housing x Genotype: F (1, 28) =0.574, p = 0.455	**Post hoc: Tuckey HSD**	SGH	A53T- vs. A53T+			p = 0.045
		MGH	A53T- vs. A53T+			p = 0.040
		A53T-	MGH vs. SGH			p = 0.999
		A53T+	MGH vs. SGH			p = 0.680
				**U**	**Z**	**p**
t-test for independent samples	C57BL/6J vs.	SGH	A53T -	27.5	0.420	0.674
			A53T +	6.5	2.626	0.008
		MGH	A53T -	24.5	0.735	0.462
			A53T +	5	2.783	0.005

Figure 2B

**Two-way ANOVA:** Housing: F (1, 28) = 0.600, p = 0.445; Genotype: F (1, 28) =12.283, p = 0.002; Housing x Genotype: F (1, 28) =0.997, p = 0.327	**Post hoc: Tuckey HSD**	SGH	A53T- vs. A53T+			p = 0.001
		MGH	A53T- vs. A53T+			p = 0.067
		A53T-	MGH vs. SGH			p = 1.000
		A53T+	MGH vs. SGH			p = 0.333
				**t**	**df**	**p**
t-test for independent samples	C57BL/6J vs.	SGH	A53T -	0.979	14	0.344
			A53T +	4.226	14	0.001
		MGH	A53T -	0.851	14	0.409
			A53T +	5.148	14	<0.001

Supplementary material

Figure 2C

Test		Group	Comparison	p
**Two-way ANOVA:**<br>Housing: F (1, 28) = 1.516, p = 0.228<br>Genotype: F (1, 28) =24.051, p <0.001<br>Housing x Genotype: F (1, 28) =1.437, p = 0.241	**Post hoc: Tuckey HSD**	SGH	A53T- vs. A53T+	p = 0.018
		MGH	A53T- vs. A53T+	p = 0.307
		A53T-	MGH vs. SGH	p = 0.999
		A53T+	MGH vs. SGH	p = 0.599

				**t**	**df**	**p**
t-test for independent samples	C57BL/ 6J vs.	SGH	A53T -	-0.290	14	0.776
			A53T +	4.034	14	0.001
		MGH	A53T -	0.086	14	0.933
			A53T +	1.512	14	0.153

Figure 2D

Test		Group	Comparison	p
**Two-way ANOVA:**<br>Housing: F (1, 28) =2.500, p = 0.125<br>Genotype: F (1, 28) =16.912, p < 0.001<br>Housing x Genotype: F (1, 28) =2.435, p = 0.130	**Post hoc: Tuckey HSD**	SGH	A53T- vs. A53T+	p = 0.002
		MGH	A53T- vs. A53T+	p = 0.292
		A53T-	MGH vs. SGH	p = 1.000
		A53T+	MGH vs. SGH	p = 0.142

				**t**	**df**	**p**
t-test for independent samples	C57BL/ 6J vs.	SGH	A53T -	-0.006	14	0.995
			A53T +	3.539	14	0.003
		MGH	A53T -	-0.024	14	0.981
			A53T +	1.948	14	0.072

Figure 2E

Test		Group	Comparison	p
**Two-way ANOVA:**<br>Housing: F (1, 28) =1.999, p = 0.168<br>Genotype: F (1, 28) =10.487, p = 0.003<br>Housing x Genotype: F (1, 28) =0.712, p = 0.406	**Post hoc: Tuckey HSD**	SGH	A53T- vs. A53T+	p = 0.035
		MGH	A53T- vs. A53T+	p = 0.346
		A53T-	MGH vs. SGH	p = 0.977
		A53T+	MGH vs. SGH	p = 0.397

				**t**	**df**	**p**
t-test for independent samples	C57BL/ 6J vs.	SGH	A53T -	0.992	14	0.338
			A53T +	3.487	14	0.004
		MGH	A53T -	0.024	14	0.841
			A53T +	1.667	14	0.118

Supplementary material

Figure 2F						
				U	Z	p
Mann Whitney U test		SGH	A53T- vs. A53T+	15	1.732	0.083
		MGH	A53T- vs. A53T+	14	1.785	0.074
		A53T-	MGH vs. SGH	29	0.262	0.793
		A53T+	MGH vs. SGH	23	0.840	0.401
				U	Z	p
Mann Whitney U test	C57BL/ 6J vs.	SGH	A53T -	24.5	0.735	0.462
			A53T +	10	2.257	0.024
		MGH	A53T -	30.5	0.105	0.916
			A53T +	16	1.628	0.104

Supplementary table S3: QUESTIONNAIRE FOR MONITORING STRESS IN LABORATORY ANIMALS

STRESS ASSESSMENT				
**Parameter**	**Animal (ID)**	**Score**	**Date/Time**	**Date/Time**
Appearance	Normal	0		
	Lack of grooming (hygienic behavior)	1		
	Rough coat, nasal discharge	2		
	Piloerection, hunching	3		
Food and Water Intake	Normal	0		
	Insufficient, body weight decreases by 5%	1		
	Insufficient, body weight decreases by 10–15%	2		
	Absence of food/water intake	3		
Clinical markers	Normal body temperature, heart rate, and respiratory rate	0		
	Mild changes in body temperature, heart rate, and respiratory rate	1		
	Temp ±1°C, heart/respiratory rate deviation by 30%	2		
	Temp ±2°C, heart/respiratory rate deviation by 50% or more	3		
Natural Behaviors	Normal behavior	0		
	Slight behavioral changes	1		
	Low mobility, altered movement, social withdrawal	2		
	Vocalization, self-mutilation, unusual calmness, drowsiness, absence of vocalization	3		
Provoked Behavior	Normal response	0		
	Slight depression or hypo- /hyperactivity	1		
	Moderate change in expected reaction to provocation	2		
	Pre-comatose state, non-responsiveness, exaggerated hypo- or hyper-reactivity	3		
Scoring Note:	If a score of 3 appears more than once, add extra points for each score of 3 → **+2 to +5**	2-5		
	**TOTAL SCORE:**	0-20		
**Stress Assessment Scale:**				

Supplementary material

0–4: Normal<br>5–9: Requires administration of analgesics or other treatment<br>10–14: Suffering, experiment needs to be stopped or adjusted

**Supplementary Table S4: Ear markings / Mice ID**	
Disclaimer: All ear markings were made around PND 21, well before the onset of Behavioral Assessment<br>Notice: Mice IDs (a combination of the following ear markings and cage numbers) are standardly used by experimenters to prevent bias when working with different groups of animals, i.e. they are blinded to mice genotype/group.	
NO	No ear markings
PLU	Hole in left ear
PDU	Hole in right ear
POU	Hole in both ears
OLU	Cut off top part of left ear
ODU	Cut off top part of right ear
OOU	Cut off the top part of both ears
RLU	Line cut in left ear
RDU	Line cut in right ear
ROU	Line cut in both ears
PLU+ODU	Hole in left ear and cut off top part of right ear
PDU+OLU	Hole in right ear and cut off the top part of left ear
PLU+RDU	Hole in left ear and line cut in right ear
PDU+RLU	Hole in right ear and line cut in left ear
OLU+RDU	Cut off top part of left ear and line cut in right ear
ODU+RLU	Cut off top part of right ear and line cut in left ear
POU+RLU	Hole in both ears and line cut in left ear
POU+RDU	Hole in both ears and line cut in right ear
OOU+RLU	Cut off the top part of both ears and line cut in left ear
OOU+RDU	Cut off the top part of both ears and line cut in left ear
OOU+PLU	Cut off the top part of both ears and hole in left ear
OOU+PDU	Cut off the top part of both ears and hole in right ear

ETHICAL STATEMENT DOCUMENT

Disclaimer: In accordance with the 3R standards a minimum of animals required for valid behavioral assessment (8 per group) was approved. Groups of animals were randomly assigned to experimental groups

Supplementary material

ИНСТИТУТ ЗА БИОЛОШКА ИСТРАЖИВАЊА
„СИНИША СТАНКОВИЋ"
БЕОГРАД

Примљено: 20.03.2023.

Орг. јед.	Број	Прилог	Вредн.
01	559		

Република Србија
МИНИСТАРСТВО ПОЉОПРИВРЕДЕ,
ШУМАРСТВА И ВОДОПРИВРЕДЕ
-УПРАВА ЗА ВЕТЕРИНУ-
Број: 323-07-02149/2023-05
Датум: 10.03.2023. године
Београд

Решавајући по захтеву Института за биолошка истраживања "Синиша Станковић", ул. Булевар Деспота Стефана 142, Београд, за издавање Решења о одобрењу спровођења огледа на животињама, на основу члана 34. Закона о добробити животиња ("Службени гласник РС" бр. 41/09), члана 136. Закона о општем управном поступку ("Службени гласник РС" број 18/2016 и 95/18 - аутентично тумачење), члана 23. став 2. Закона о државној управи ("Службени гласник РС" број 79/05 и 101/07, 95/2010 и 99/2014, 47/18 и 30/18 и други закон) и Решења о овлашћењу министра пољопривреде, шумарства и водопривреде Републике Србије бр: 119-01-4/36/2022-09 од 28.12.2022. године, в.д. директора Управе за ветерину, Доц. др Милош Петровић, доноси:

РЕШЕЊЕ

УСВАЈА СЕ захтев Института за биолошка истраживања "Синиша Станковић", ул. Булевар Деспота Стефана 142, Београд, и издаје се Решење о одобрењу спровођења огледа на животињама под називом:

"Испитивање хиперактивности, емоционалних и меморијских карактеристика А53Т трансгених мужјака мишева пре појаве моторичког дефицита".

Образложење

Институт за биолошка истраживања "Синиша Станковић", ул. Булевар Деспота Стефана 142, Београд, обратио се овом министарству дана 08.03.2023. године, захтевом за издавање Решења о одобрењу спровођења огледа на животињама под називом: "Испитивање хиперактивности, емоционалних и меморијских карактеристика А53Т трансгених мужјака мишева пре појаве моторичког дефицита".

Подносилац пријаве је Министарству, уз захтев, доставио документацију прописану Законом о добробити животиња ("Службени гласник РС' бр. 41/09) и Правилником о условима за упис у регистар за огледе на животињама и садржини и начину вођења тог Регистра, програму обуке о добробити огледних животиња обрасцу захтева за одобрење спровођења огледа на животињама, начину неге, поступања и лишавању живота огледних животиња, као и садржини и начину вођења евиденције о држању, репродукцији, промету, односно спровођењу огледа на животињама ("Службени гласник РС', бр. 39/10). Саставни део документације чини и мишљење Етичке комисије.

Supplementary material

Приликом решавања по захтеву, на основу увида у приложену документацију, утврђено је да су се стекли услови за доношење решења као у диспозитиву, сходно одредбама члана 136. Закона о општем управном поступку ("Службени гласник РС" број 18/2016 и 95/18 - аутентично тумачење).

Поука о правном средству: Ово решење је коначно у управном поступку. Против овог решења се може покренути управни спор подношењем тужбе Управном суду Србије у року од 30 дана од дана пријема решења.

На основу члана 18., тачка 1. ЗОАТ-а, подносилац пријаве је ослобођен плаћања таксе.

РЕПУБЛИКА СРБИЈА МИНИСТАРСТВО ПОЉОПРИВРЕДЕ ШУМАРСТВА ... БЕОГРАД

В.д. ДИРЕКТОРА

Доц. др Милош Петровић

Доставити:

1. Институт за биолошка истраживања "Синиша Станковић", Булевар Деспота Стефана 142, 11060 Београд
2. Реп. ветеринарска инспекција, Велислава Вуловића 1А/4, 11000 Београд
3. Евиденција
4. Архива
